# A Search for Novel *Legionella pneumophila* Effector Proteins Reveals a Strain Specific Nucleotropic Effector

**DOI:** 10.3389/fcimb.2022.864626

**Published:** 2022-05-31

**Authors:** Inês P. Monteiro, Sofia Sousa, Vítor Borges, Paulo Gonçalves, João Paulo Gomes, Luís Jaime Mota, Irina S. Franco

**Affiliations:** ^1^ UCIBIO– Applied Molecular Biosciences Unit, Department of Life Sciences, NOVA School of Science and Technology, NOVA University Lisbon, Caparica, Portugal; ^2^ Núcleo de Bioinformática, Instituto Nacional de Saúde Dr. Ricardo Jorge, Lisboa, Portugal; ^3^ Laboratório Nacional de Referência de Legionella, Instituto Nacional de Saúde Dr. Ricardo Jorge, Lisboa, Portugal; ^4^ Associate Laboratory i4HB - Institute for Health and Bioeconomy, NOVA School of Science and Technology, NOVA University Lisbon, Caparica, Portugal

**Keywords:** *Legionella pneumophila*, infection, outbreak strain, type 4 secretion system (T4SS), Icm/Dot effectors, nucleomodulin, homolog

## Abstract

*Legionella pneumophila* is an accidental human pathogen that causes the potentially fatal Legionnaires’ disease, a severe type of pneumonia. The main virulence mechanism of *L. pneumophila* is a Type 4B Secretion System (T4SS) named Icm/Dot that transports effector proteins into the host cell cytosol. The concerted action of effectors on several host cell processes leads to the formation of an intracellular Legionella-containing vacuole that is replication competent and avoids phagolysosomal degradation. To date over 300 Icm/Dot substrates have been identified. In this study, we searched the genome of a *L. pneumophila* strain (Pt/VFX2014) responsible for the second largest *L. pneumophila* outbreak worldwide (in Vila Franca de Xira, Portugal, in 2014) for genes encoding potential novel Icm/Dot substrates. This strain Pt/VFX2014 belongs to serogroup 1 but phylogenetically segregates from all other serogroup 1 strains previously sequenced, displaying a unique mosaic genetic backbone. The ability of the selected putative effectors to be delivered into host cells by the T4SS was confirmed using the TEM-1 β-lactamase reporter assay. Two previously unknown Icm/Dot effectors were identified, VFX05045 and VFX10045, whose homologs Lpp1450 and Lpp3070 in clinical strain *L. pneumophila* Paris were also confirmed as T4SS substrates. After delivery into the host cell cytosol, homologs VFX05045/Lpp1450 remained diffused in the cell, similarly to Lpp3070. In contrast, VFX10045 localized to the host cell nucleus. To understand how VFX10045 and Lpp3070 (94% of identity at amino acid level) are directed to distinct sites, we carried out a comprehensive site-directed mutagenesis followed by analyses of the subcellular localization of the mutant proteins. This led to the delineation of region in the C-terminal part (residues 380 to 534) of the 583 amino acid-long VFX10045 as necessary and sufficient for nuclear targeting and highlighted the fundamental function of the VFX10045-specific R440 and I441 residues in this process. These studies revealed a strain-specific nucleotropism for new effector VFX10045/Lpp3070, which anticipates distinct functions between these homologs.

## Introduction

*Legionella pneumophila* is a facultative intracellular pathogen responsible for Legionnaires’ Disease, a severe type of pneumonia which can develop after the inhalation of contaminated aerosols and infection of lung macrophages (Newton et al., 2010). *Legionella pneumophila* is found ubiquitously in freshwater habitats where it parasitizes amoebae, its environmental host. In man-made habitats, these interactions with the surrounding environmental microbiota provide a source of dissemination and trigger virulence traits that aid in subsequent infection of human hosts (Molmeret et al., 2005). Its ability to thrive intracellularly in amoebae or in human lung macrophages is based on the capacity of *L. pneumophila* to inject over 300 bacterial effector proteins into host cells. After *L. pneumophila* enters the phagocytic host cell, it resides within a remodeled, protective compartment known as the Legionella-containing vacuole (LCV) that escapes lysosomal degradation. Formation of the LCV requires a Type 4 Secretion System named Icm/Dot that delivers bacterial effector proteins into the host cell cytosol (Marra et al., 1992; Berger and Isberg, 1993). These effectors target a multitude of host cell components to subvert numerous eukaryotic processes such as cytoskeleton dynamics, vesicle trafficking, apoptosis, transcription or translation (Best and Kwaik, 2018; Omotade and Roy, 2021).

Bacterial nucleomodulins are an emerging group of effectors that after being delivered into eukaryotic host cells enter the nucleus and modulate processes therein, namely gene expression and immune response (Bierne and Cossart, 2012; reviewed in Hanford et al., 2021) Nucleomodulins have been identified in diverse pathogenic bacteria, such as in phytopathogens like *Agrobacterium* and *Xanthomonas*, or animal/human pathogens such as *Mycobacterium, Chlamydia, Salmonella or Yersinia*. These nuclear-targeted effectors use a variety of mechanisms, which include acting as transcription factors [*Ehrlichia chaffeensis* TRP120; (Klema et al., 2018)], mediating the integration of foreign DNA into the host cell genome [*Agrobacterium tumefaciens* VirD2; (Mysore et al., 1998)], altering the nuclear envelope (*Chlamydia psittaci* SINC; (Mojica et al., 2015), modifying histones (*C. trachomatis* NUE; (Pennini et al., 2010) or interacting with the transcription complex (*Escherichia coli* NleG; Valleau et al., 2018). *L. pneumophila* delivers into host cells at least four known nucleomodulins: LegAS4/RomA (Li et al., 2013; Rolando et al., 2013), SnpL (Schuelein et al., 2018), AnkH (Von Dwingelo et al., 2019) and Lpg2936 (Abd El Maksoud et al., 2020). LegAS4 and RomA are homologous nucleomodulins from strains Philadelphia-1 and Paris, respectively. They are histone lysine methyltransferases that modify different lysine residues on histone H3. RomA leads to a global repression of transcription, particularly of host innate immunity genes (Rolando et al., 2013), whereas LegAS4 causes an increased transcription of host rDNA genes *via* interaction with HP1α (Heterochromatin protein 1 α) (Li et al., 2013). SnpL interferes with the host RNA polymerase II leading to a global gene activation in macrophages (Schuelein et al., 2018), whereas AnkH reprograms transcription *via* modulation of the activity of 7SK small nuclear ribonucleoprotein (snRNP) complex, resulting in enhanced permissiveness to *L. pneumophila* (Von Dwingelo et al., 2019). Another effector, Lpg2936, contributes to bacterial intracellular replication by epigenetic modification of the promoter regions of autophagy genes (Abd El Maksoud et al., 2020).

Legionnaires’ disease outbreaks occur frequently, and the second largest outbreak worldwide occurred in Portugal in 2014. This outbreak was caused by a new *L. pneumophila* strain, *L. pneumophila* PtVFX/2014, and originated the first reported case of person-to-person transmission (Correia et al., 2016). *L. pneumophila* PtVFX/2014 belongs to serogroup 1 (Sg1) but analysis of its genome revealed a phylogenetic divergence from the most widely studied *L. pneumophila *Sg1 strains, such as *L. pneumophila* Philadelphia-1 or *L. pneumophila* Paris (Borges et al., 2016). In fact, *L. pneumophila* PtVFX/2014 has shown to belong to the *L. pneumophila* subspecies *fraseri* and to have a strong horizontal gene transfer inheritance, involving multiple virulence factors (Borges et al., 2016). Thus, this strain may have acquired specific traits that allow it to adapt and persist in environments and be transmitted to humans.

In this work, we identified two novel effectors encoded in the genome of the outbreak strain *L. pneumophila* Pt/VFX2014. These effectors, VFX05045 and VFX10045, have homologs in other *L. pneumophila* strains, with the highest similarity displayed by *L. pneumophila* Paris proteins Lpp1450 and Lpp3070. Homologs VFX05045/Lpp1450 showed an identical cytosolic localization, during infection of macrophages and when ectopically expressed in mammalian cells. However, a contrasting distribution was observed for homologs VFX10045 and Lpp3070, with VFX10045 being targeted to the host cell nucleus while Lpp3070 showed a cytosolic localization. These results anticipate strain-specific roles for the *L. pneumophila* effector VFX10045, a phenomenon that may contribute to the functionally diversified effectorome of this pathogen.

## Materials and Methods

### Strains and Media

*L. pneumophila* and *E. coli* strains (listed in **Table S1**) used in this work were grown as previously described (Shohdy et al., 2005; Franco et al., 2012). For construction of deletion mutants Δ*lpp1450*, Δ*lpp3070* and Δ*vfx10045*, *L. pneumophila* strains were transformed with DNA fragments containing a kanamycin resistance cassette (Kan^R^) flanked by ~1kb regions upstream or downstream from the corresponding genes. To facilitate the attainment of this DNA fragment, each of these three regions (“upstream”, “Kan^R^” and “downstream”) was independently amplified by PCR using oligos with restriction sites that allowed their progressive cloning at high copy number plasmid pUC18 (Thermo Fisher Scientific). The PCR product subsequently used for transformation was then obtained by amplification with the flanking primers (see **Table S2**).

### Plasmids and Oligonucleotides

Plasmids and oligonucleotides used in this study are listed, respectively, in Tables S2 and S3, as well as details of how plasmids were constructed. For general cloning procedures, restriction enzymes (Thermo Fisher Scientific), T4 DNA ligase (Thermo Fisher Scientific), Phusion polymerase (Thermo Fisher Scientific) were used according to the manufacturer’s instructions. For site-directed mutagenesis, amino acid substitutions in VFX10045 were made by overlap PCR with oligonucleotides carrying appropriate modified nucleotides. The accuracy of the nucleotide sequence of the constructs was confirmed by DNA sequencing.

### Mammalian Cell Culture, Transfections and Infections

CHO FcγRII cells (Joiner et al., 1990) were grown in Dulbecco’s Modified Eagle Medium (DMEM; Corning) and 10% (v/v) heat-inactivated fetal bovine serum (FBS; Thermo Fisher Scientific), at 37°C in a 5% (v/v) CO_2_ incubator. For immunoblot experiments, CHO cells were seeded on 24-well plates at 1x10^5^ cells/well, for microscopy experiments at 5x10^4^ cells/well. CHO cells were transfected using the jetPEI™ reagent (Polyplus) according to manufacturer’s protocol for 24 hours. THP-1 human monocyte-like cells were grown in RPMI 1640 (Thermo Fisher Scientific) and 10% FBS (Thermo Fisher Scientific), 1 mM L-Glutamine (Thermo Fisher Scientific), 10 mM HEPES (Thermo Fisher Scientific), Sodium Pyruvate 1 mM (Thermo Fisher Scientific) and 0.05 mM β-mercaptoethanol at 37°C in a 5% (v/v) CO_2_ incubator. Infection of THP-1 cells was carried out as described below. For infection experiments, THP-1 cells were seeded at 5x10^5^ cells/well and allowed to differentiate for 24 hours in the presence of phorbol 12-myristate 13-acetate (PMA), after which they were incubated with fresh RPMI for additional 24 hours.

### Immunofluorescence Microscopy and Quantification of Nuclear Localization

Transfected CHO cells or infected THP-1 cells were fixed and permeabilized for immunofluorescence microscopy as described previously, using Triton X 0.1% for cell permeabilization. For labelings, we used mouse anti-FLAG (Sigma; 1:200), mouse anti-myc (Calbiochem; 1:200), rat anti-HA (Sigma; 1:200), followed by appropriate fluorophore-conjugated anti-mouse antibodies (Jackson ImmunoResearch; 1:200). 4′,6-Diamidino-2-phenylindole (DAPI; 1:30.000) was used to label DNA, and actin staining was carried out by incubating CHO cells with Phalloidin-Alexa555 (Thermo Fisher Scientific, 1:200) during 30 min. Images were acquired on an Axio Imager D2 (Zeiss) and processed with ZEN or Fiji software. For each enhanced GFP (EGFP) fusion protein, the proportion of protein in the nucleus was determined by calculating the ratio between the average GFP fluorescence in the nucleus and the average GFP fluorescence in the cytosol. Quantification of these values was made in Fiji, using the DAPI stain to delineate the nucleus and the F-actin stain for the cell outline. For each protein construct, fluorescence was quantified for at least 60 cells from 3 independent experiments. Statistical significance was assessed with Student’s T-test.

### Immunoblotting

For the preparation of bacterial cell extracts, *L. pneumophila* strains were grown on charcoal yeast extract (CYE) plates supplemented with appropriate antibiotics for 4-5 days at 37°C. Bacteria were patched on identical plates with isopropyl β-D-1-thiogalactopyranoside (IPTG) 1 mM and grown for additional 24 hours. From these plates, a loop of bacteria was removed and resuspended in sterile water, the optical density at 600 nm (OD_600_) measured and resuspended in appropriate volumes of SDS-PAGE loading buffer in order to obtain the same concentration of bacteria. For the preparation of mammalian cell extracts, transfected CHO cells were washed with PBS and trypsinized with 100 µl Tryple Express (Thermo Fisher Scientific), resuspended in 900 µl DMEM, collected by centrifugation at 720 g for 5 min and washed in phosphate-buffered saline (PBS) twice. The pellets were resuspended in SDS-PAGE loading buffer, boiled for 5 min and loaded for SDS-PAGE, after which gels were processed for immunoblotting using Trans-Blot Turbo Transfer System (BioRad) and 0.2 μm pore-size nitrocellulose membranes (BioRad). The following antibodies were used for immunoblotting: goat anti-GFP (SICGEN; 1:1000), mouse anti-α-Tubulin (Sigma; 1:1000), mouse anti-FLAG (Sigma-Aldrich; 1:1000), rat anti-HA (Sigma; 1:1000), rabbit anti-myc (Cell Signalling Technologies), mouse anti-TEM (QEDBiosciences Inc; 1:500), rabbit anti-*Legionella pneumophila* MOMP (1:1000) followed by appropriate horseradish peroxidase (HRP)-conjugated secondary antibodies (GE Healthcare or Jackson ImmunoResearch; 1:10.000). Immunoblot detection was done with SuperSignal West Pico ECL (Thermo Scientific) and exposure to Amersham Hyperfilm ECL (GE Healthcare).

### TEM-1 β-Lactamase Assays

Assays were performed essentially as described (Allombert et al., 2017) using a LiveBLAzer™ FRET - B/G Loading CCF2/AM kit (Thermo Fisher Scientific). *L. pneumophila* strains harboring pXDC61 derivatives encoding TEM fusions to putative effectors (wildtype or *dotA* background; see Table S1 and S2) were grown in ACES-buffered yeast extract (AYE) supplemented with 1 mM IPTG and required antibiotics at 37°C overnight with agitation. THP-1 human monocyte-like cells were differentiated on coverslips and infected for 1 hour using an MOI=50. Cells were loaded with CCF2/AM, incubated for 2 hours in the dark, washed with PBS and fixed for 20 min with 4% PFA, followed by mounting. For quantification of protein delivery (translocation) into host cells images were acquired from the DAPI and FITC channels using fixed exposure times and ZEN software (Zeiss). Background fluorescence was imaged from coverslips without cells. To obtain the ratio of blue/green fluorescence, total blue and total green fluorescence in each image was measured (Fiji software), the background blue and green fluorescence was subtracted, and blue/green ratio calculated. Data obtained represent the mean value of blue/green ratios from three independent experiments, each with 11 analysed images for each strain.

### Intracellular Replication Assays in THP-1 Macrophages

THP-1 cells were seeded at 5x10^5^ cells/well in 24-well plates and differentiated as described above. *L. pneumophila* strains were grown on CYE plates for 4-5 days at 37°C. From these plates, a loop of bacteria was removed and resuspended in sterile water, the OD_600_ measured and appropriate dilutions made to obtain an infection stock at 5x10^5^ bacteria/ml. A volume of 100 µl of the infection stock was added to each well, corresponding to a multiplicity of infection of 0.1. Infection was synchronized by centrifuging at 800 g for 10 min. Infection was allowed to occur for 1.5 hours in a CO_2_ incubator, after which cells were washed with PBS and RPMI containing gentamicin (100 µg/ml) was added to each well. Following an additional incubation for 1 hour, cells were washed with PBS and fresh RPMI was added (T_0_). To calculate CFUs at appropriate time-points, lysis was carried out in the following manner: cells were washed with PBS, incubated with 1 ml of sterile water at room temperature for 15 min and vigorously resuspended. The lysate was subjected to appropriate serial dilutions and 100 µl plated on CYE, incubated for 5 days at 37°C and colony forming unites (CFUs) counted.

### *In Silico* Search for Icm/Dot Putative Substrates/Effectors in the Novel Strain *L. pneumophila* Pt/VFX2014

To search for previously unidentified Icm/Dot substrates/effectors in the novel strain *L. pneumophila* Pt/VFX2014 (draft genome accession available from https://www.ncbi.nlm.nih.gov/nuccore/LORH00000000.1), we first screened the Pt/VFX2014 predicted protein sequences (https://sra-download.ncbi.nlm.nih.gov/traces/wgs03/wgs_aux/LO/RH/LORH01/LORH01.1.fsa_aa.gz) using the web EffectiveDB platform (https://effectors.csb.univie.ac.at/; accessed on 25-26 July 2016) (Jehl et al., 2011; Eichinger et al., 2016), which provides several online tools for detecting putative bacterial effectors and predicting eukaryotic-like domains likely to interact with host proteins. Specifically, the following tools (and settings) were applied: i) T4SEpre (*minimal score: 0.9999*), which predicts Type IV secreted proteins based on amino acid composition in C-termini; ii) EffectiveELD (*minimal score: 5*), which predicts secreted proteins based on eukaryotic-like domains; iii) Predotar (*model: human/animal*), which predicts subcellular localization of secreted proteins in the host cell; and *iv*) EffectiveS346 (*enabled*), which predicts Type III, IV, VI secretion systems for protein sequences from (nearly) complete genomes. From the 128 out of the 2991 proteins predicted as potential T4SS effectors by T4SEpre, 23 and two proteins also had a hit in Predotar and EffectiveELD, respectively. Of these 25 candidates, only four (PtVFX2014_03805, PtVFX2014_06065, PtVFX2014_12350 and PtVFX2014_13425) have not previously been described as putative effectors in literature after cross-checking against a custom database of the repertoire of known Dot/Icm substrates in Pt/VFX2014 strain (summarized in Supplementary Table 2 in Borges et al., 2016 and additional literature (Zhu et al., 2011). In parallel to this *in silico* prediction, we navigated through the Pt/VFX2014 genome annotation to identify (clusters of) genes (not previously unidentified as Icm/Dot effectors) that are encoded adjacently to known T4SS substrates. From this search, we selected these additional candidates: i) PtVFX2014_05045 and PtVFX2014_05055 (absent in Philadelphia-1 strain, but encoded in a region - *lpg1491-lpg1496* - encoding known T4SS substrates); ii) PtVFX2014_09510 (absent in Philadelphia-1 strain, but encoded adjacently to the T4SS substrate *lpg0096/ceg4*; and, *iii*) PtVFX2014_10045 (absent in Philadelphia-1 strain, but encoded adjacently to the T4SS substrated *lpg2999 and lpg3000*). In summary, eight candidate proteins (PtVFX2014_03805, PtVFX2014_06065, PtVFX2014_12350, PtVFX2014_13425, PtVFX2014_05045 [GenBank KZX34370.1], PtVFX2014_05055, PtVFX2014_09510 and PtVFX2014_10045 [GenBank KZX33632.1]) were selected to proceed to experimental assays. For the sake of simplicity, the prefix of GenBank locus tags “PtVFX2014_” is referred as “VFX” throughout the text.

## Results

### *L. pneumophila* Pt/VFX2014 Encodes Two New Substrates of the Icm/Dot Secretion System, Effectors VFX05045 and VFX10045

**Figure 1:**
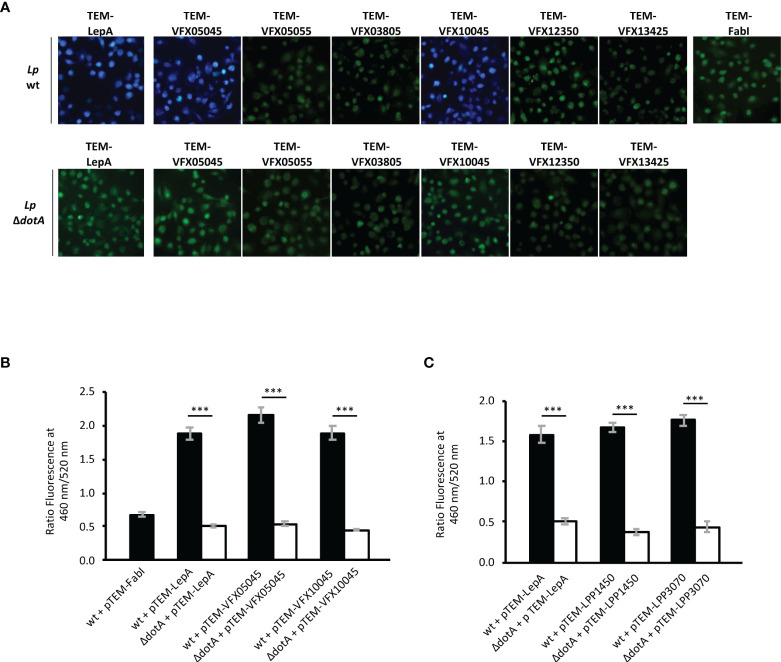
New protein substrates of the Icm/Dot type 4 secretion system from strains *L. pneumophila* Pt/VFX2014 and *L. pneumophila* Paris. Translocation assays were performed in strains harboring plasmid encoded TEM-1 β-lactamase fusion proteins: FabI, non-translocated protein; LepA, translocated protein; and Icm/Dot candidate substrates VFX05045, VFX05055, VFX03805, VFX10045, VFX12350 and VFX13425. Plasmids encoding the fusion proteins were introduced both in an Icm/Dot^+^ background (wildtype strain *L. pneumophila* JR32 in A and B, or *L. pneumophila* Paris in C) or Icm/Dot^-^ background (corresponding *dotA* derivatives). **(A)** Merged images of fluorescence emission acquired at 460 nm (blue channel) and 520 nm (green channel). **(B, C)** Total fluorescence obtained for each channel was quantified using FIJI software (see Materials and Methods) and translocation expressed as a ratio of blue/green fluorescence (460/520 nm). Error bars represent the standard error of the mean of three independent biological repeats, each with at least 10 samples. Statistical analysis was made using Student’s t-test (***, p<0.001).

Based on *in silico* predictions (as detailed in Materials and Methods), eight proteins of the novel strain *L. pneumophila* Pt/VFX2014 were selected as candidate Icm/Dot substrates: VFX03805, VFX05045, VFX05055, VFX06065, VFX09510, VFX12350, VFX13425 and VFX10045. To assess if these putative effectors were indeed delivered (i.e., translocated) into host cells by the Icm/Dot T4SS secretion system, we used the TEM-1 β-lactamase FRET-based reporter system (Allombert et al., 2017). In this methodology, fusion proteins containing N-terminal TEM-1 are produced in *L. pneumophila* under the control of the *Ptac* IPTG-inducible promoter. Their transport into to the cytosol of macrophages previously loaded with the compound CCF2/AM should lead to its cleavage by the TEM-1 β-lactamase, resulting in the emission of blue fluorescence which contrasts to the intrinsic green fluorescence of CCF2/AM. Plasmids encoding fusion proteins of TEM-1 to the eight putative effectors (**Table S2**) were transformed into *L. pneumophila* JR32 wild-type, or into an Icm/Dot-deficient derivative (*dotA* mutant; used as negative control for type 4 secretion mediated transport into host cells). As additional controls, we used strains expressing TEM-1 fusions to FabI, a non-translocated *L. pneumophila* protein, or to the characterized effector LepA (de Felipe et al., 2008). Production of proteins TEM-VFX06065 and TEM-VFX09510 was not detected by immunoblotting, thus the analyses were performed with the remaining six candidates. Production of these six TEM-1 fusion proteins by *L. pneumophila* was tested by immunoblot using an anti-TEM antibody (**Figure S1A**). Infection of THP-1 macrophages was carried out for 2 hours, after which CCF2/AM was added and cleavage of the compound allowed to occur for 1 hour. The presence of the candidate effectors in the macrophage cytosol was then assessed by fluorescence microscopy (**Figure 1A**). Only *L. pneumophila* strains expressing protein fusions TEM-1-VFX05045 and VFX10045 gave rise to a blue fluorescence in macrophages, identical to the TEM-LepA positive control. Determination of total blue and green fluorescence and calculation of the blue/green (B/G) ratio allowed the quantification of translocated TEM-1 fusion proteins for each strain. Comparison of the B/G ratio obtained for the positive and negative controls confirmed the translocation of VFX05045 and VFX10045 *via* the Icm/Dot secretion system (**Figure 1B**). Therefore, proteins VFX05045 and VFX10045 encoded by the outbreak strain *L. pneumophila* Pt/VFX2014 are newly discovered T4SS substrates transported into the host cell cytosol during infection.

### Homologs of VFX05045 and VFX10045 in *L. pneumophila* Paris, Lpp1450 and Lpp3070, Are Also Icm/Dot Substrates

**Figure 2:**
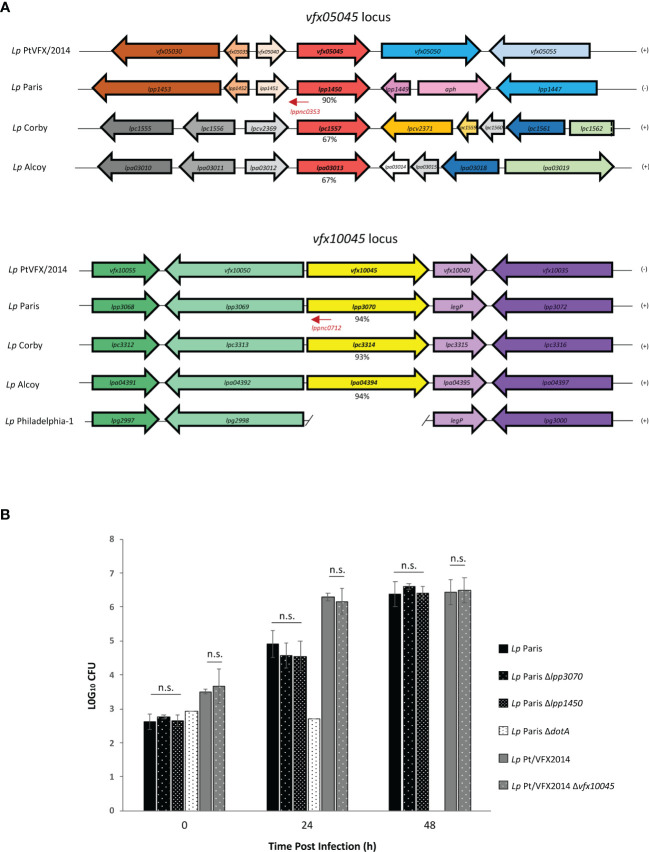
Effector encoding genes *vfx10045/lpp3070* and *vfx05045/lpp1450* are present in the genomes of other *L. pneumophila* strains and dispensable for intracellular replication in THP-1 macrophages. **(A)** Genetic organization of the *vfx05045* and *vfx10045* loci in *L. pneumophila* outbreak strain and of homologs in *L. pneumophila* Paris, Corby, Alcoy and Philadelphia-1. Identical colors reflect amino acid identity >60% at the encoded protein level. lppnc represent non-coding RNAs (LegioList database). The percentages of identity at the amino acid level of proteins *VFX05045* and *VFX10045* with homologs are indicated. **(B)** Intracellular replication asssays of *L. pneumophila* Paris and derivatives *ΔdotA, Δlpp1450* and *Δlpp3070*, and *L. pneumophila* Pt/VFX2014 and derivative *Δvfx10045* were performed by infecting THP-1 macrophages for 90 minutes with a multiplicity of infection of 0.1. To kill extracellular bacteria, cells were then incubated with RPMI containing gentamicin 100 µg/ml for 1 hour, after which cells were washed with PBS and fresh RPMI was added (T_0_). At 0, 24 and 48 h post-infection lysis was accomplished in 1 ml of sterile water at room temperature for 15 min. The lysate was subjected to appropriate serial dilutions, plated on CYE and CFUs counted. The results shown represent the average and standard deviation of 3 independent experiments, each assayed in duplicate. n.s., non-significant statistical differences relative corresponding parental strain.

A search in the genomes of other *L. pneumophila* strains revealed the presence of coding regions for homologs of VFX05045 and VFX10045 in *L. pneumophila* Paris, Alcoy and Corby, but missing in *L. pneumophila* Philadelphia-1 and in other *Legionella* sp. (**Figure 2**). The genomic region surrounding *vfx1004*5 is conserved in strains *L. pneumophila* Paris, Alcoy and Corby, and the genome of strain Philadelphia-1 contains an identical region except for the absence of the *vfx10045 locus*. In contrast, for *vfx05045* the genomic context is only partially conserved in *L. pneumophila* Paris and distinct in *L. pneumophila* Alcoy and Corby. For both *vfx05045* and *vfx10045*, their expression may be regulated by predicted antisense non-coding RNAs that target the effector transcripts, lppnc0353 and lppnc0712, respectively (Sahr et al., 2012). Evaluation of the amino acid sequence of the VFX05045 and VFX10045 homologs revealed that *L. pneumophila* Paris proteins Lpp1450 and Lpp3070 shared the highest degree of identity, 90% and 94% respectively (**Figure S2** and **Figure 2**). To confirm that Lpp1450 and Lpp3070 are also *bona fide* substrates of the Icm/Dot system, studies with TEM-1 β-lactamase fusions were performed, as described above. Using this methodology, the presence of both proteins was detected in the macrophage cytosol, showing that *L. pneumophila* Paris Lpp1450 and Lpp3070 are also Icm/Dot substrates (**Figure 1C**; **Figure S1B**).

### Lpp1450 and Lpp3070 Are Not Essential for Intracellular Multiplication of *L. pneumophila* in THP-1 Macrophages

To examine the importance of *L. pneumophila* Lpp1450 and Lpp3070 proteins for an efficient infection of macrophages, null mutants were constructed by allelic replacement with a Kan^R^ cassette. THP-1 macrophages were then infected with wild-type *L. pneumophila* Paris or derivatives Δ*dotA*, Δ*lpp3070* and *Δlpp1450*, and replication inside these cells was monitored by CFU counting at 0 h, 24 h and 48 h after infection (**Figure 2B**). The results showed no significant differences in uptake or intracellular replication resulting from deletion of either *lpp1450* or *lpp3070*. Similarly, loss of *vfx10045* did not yield any defect in this assay when compared to wild-type *L. pneumophila* Pt/VFX2014. This lack of phenotype is not surprising, as deletion of most *L. pneumophila* effector genes has no obvious repercussion on replication rate. Thus, effectors Lpp1450, Lpp3070 and VFX10045 are not essential for *L. pneumophila* replication inside macrophages.

### VFX10045 Displays a Nuclear Tropism Absent in Homolog Lpp3070

**Figure 3:**
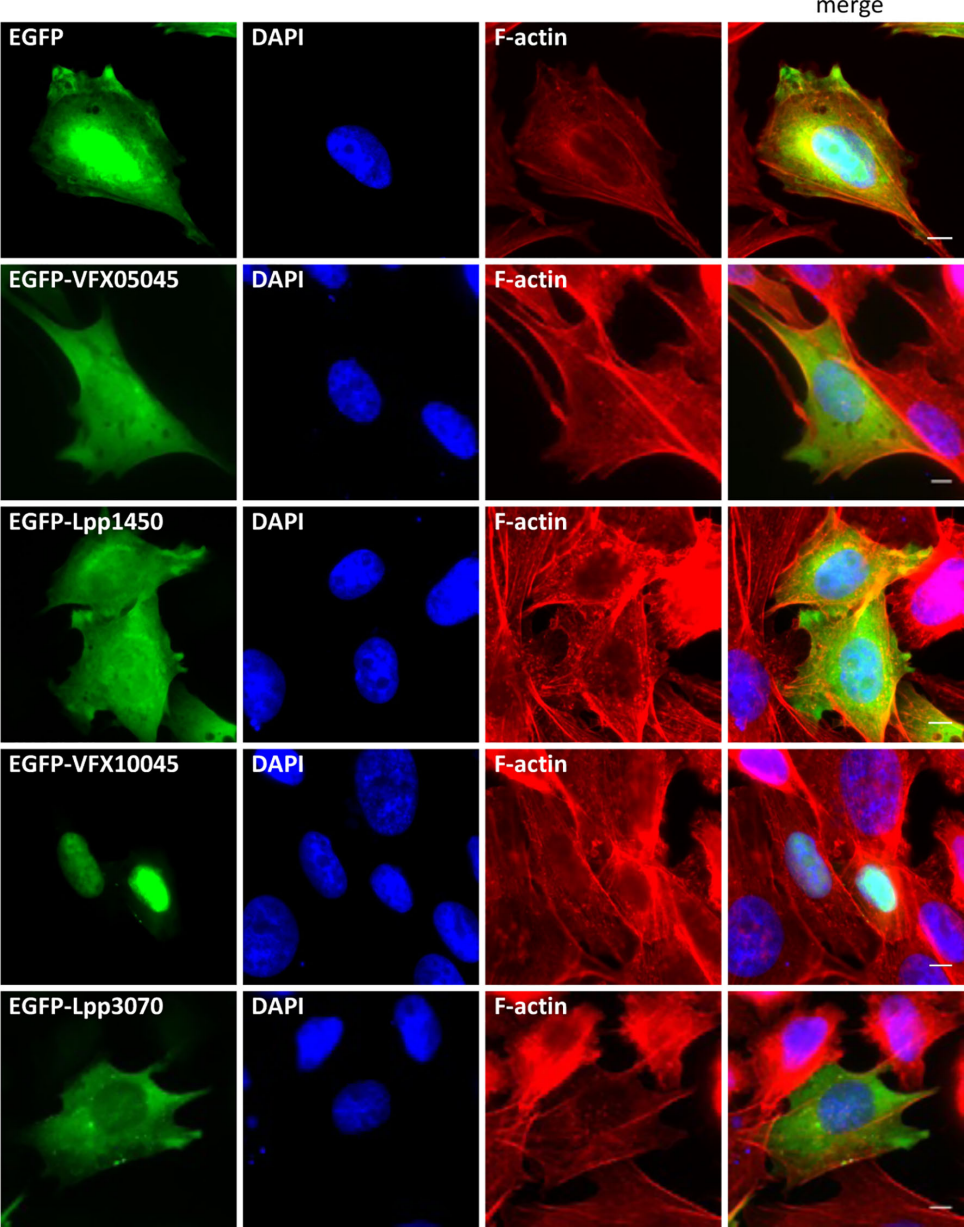
Subcellular localization of *L. pneumophila* Icm/Dot substrates orthologs VFX05045, Lpp1450, VFX10045 and Lpp3070 in CHO cells. Plasmids encoding EGFP fusions to VFX05045, VFX10045, Lpp1450 and Lpp3070 were used to transiently transfect CHO mammalian cells. Cells were fixed with 4% PFA (w/v), permeabilized with 0.1% Triton X (v/v) and labeled with Phalloidin-AlexaFluor-555 and DAPI. Scale bar, 5 µm.

To gain insight into the function of these newly discovered Icm/Dot substrates, we started by assessing their subcellular localization as EGFP fusion proteins when expressed ectopically in mammalian CHO cells, a cell line commonly used as a model for these studies. For this, plasmids encoding EGFP, EGFP-VFX05045, EGFP-Lpp1450, EGFP-VFX10045, or EGFP-Lpp3070 were used to transiently transfect CHO cells, which were further analysed by fluorescence microscopy after fixation and fluorescence staining of the nucleus and actin cytoskeleton. Localization of EGFP-VFX10045 was identical to the one displayed by cognate EGFP-Lpp1450, being both homogeneously dispersed in the cell cytosol (**Figure 3**). However, EGFP-VFX10045 showed a striking localization in the cell nucleus, which was identical when using other tags in this protein (myc-VFX10045, VFX10045-myc, and 3xFLAG-VFX10045; **Figure S3**). Surprisingly, its homolog Lpp3070 showed a different distribution pattern, being mostly spread in the cytosol with some occasional enriched patches (EGFP-Lpp3070 and Lpp3070-myc; **Figure S3**). To rule out the possibility of an artifactual localization of the fusion proteins due to their degradation or cleavage in the cell, we confirmed by western blot that they were being produced as the expected full-length versions (approximately 68 kDa for VFX05045/Lpp1450 and 95 kDa for VFX10045/Lpp3070; **Figures S1D**, **S3B**).

**Figure 4:**
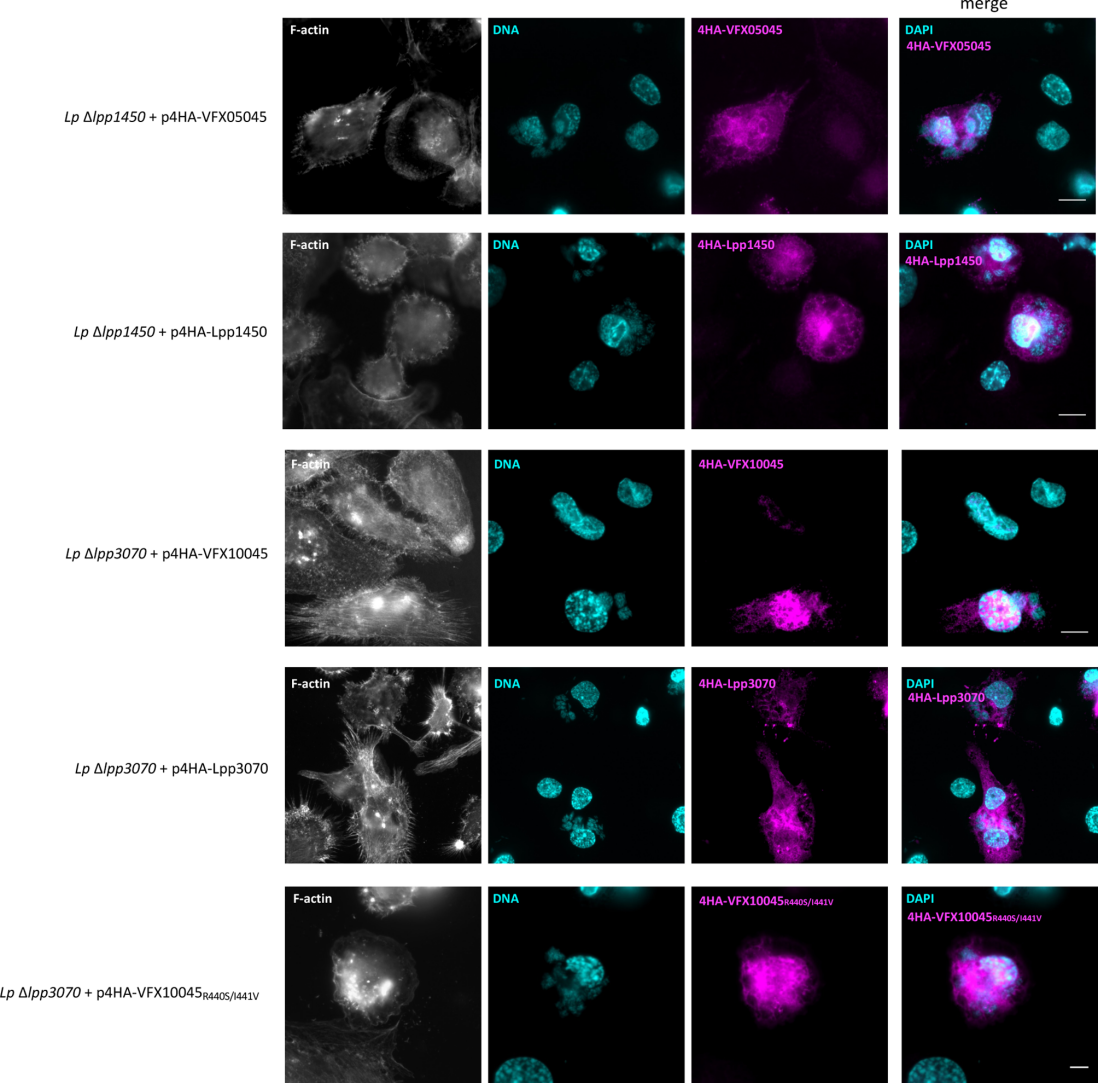
Subcellular localization of *L. pneumophila *effectors VFX05045, Lpp1450, VFX10045 and Lpp3070 during infection of THP-1 cells. THP-1 cells were infected with the indicated *L. pneumophila* Paris derivative strains for 24 hours with multiplicities of infection of 5. Cells were fixed with 4% PFA (w/v) and permeabilized with 0.1% Triton X100 (v/v). F-actin was labeled with Phalloidin-AlexaFluor-488 (shown in white), DNA was labeled with DAPI (in cyan) and translocated HA-tagged effectors were labeled with primary anti-HA (rat) and secondary anti-rat-RRX antibodies (in magenta). Scale bar, 10 µm.

To analyse the localization of these proteins in the context of macrophage infection, *L. pneumophila* Paris derivative strains were used that expressed plasmid-encoded fusions of VFX05045, VFX10045, Lpp1450 and Lpp3070 to an N-terminal 4xHA tag. Infection of THP-1 macrophages was carried out for 24 hours, a time-point at which all translocated effectors were clearly visualized within host cells. After fixation and permeabilization, fluorescence labeling was performed for F-actin, DNA (cell nucleus and bacteria) and the 4HA-tagged proteins. Homologs VFX05045 and Lpp1450 dispersed essentially throughout the host cell cytosol (**Figure 4**, upper panels), in agreement with what was observed in the transfection experiments (**Figure 3**). 4HA-VFX10045 accumulated in the nucleus of most infected cells, while in contrast homolog 4HA-Lpp3070 essentially distributed in the cytosol with occasional enrichments, namely in cortical cell-cell contact regions (**Figure 4**). All four 4HA-tagged proteins migrated on SDS-PAGE according to their expected molecular mass, when analyzed by immunoblotting (**Figure S1C**). Taken together, subcellular localization analyses of these *L. pneumophila* Icm/Dot substrates were consistent in transfection and infection experiments and revealed a nuclear tropism for VFX10045.

### Targeting of VFX10045 to the Host Cell Nucleus Is Mediated by a Region Comprised Between Amino Acid Residues S_380_ and Q_534_

Transport of proteins into the eukaryotic nucleus may be accomplished by passive diffusion or by transport factors that recognize specific nuclear localization sequence(s) (NLSs). The migration pattern observed in SDS-PAGE for the previously analysed tagged versions of effector VFX10045 (**Figures S1** and **S3B**) is in accordance with the predicted molecular mass of ~68 kDa for the untagged protein, which precludes its localization to the nucleus *via* diffusion, as this does not occur in macromolecules over 50-60 kDa.

**Figure 5:**
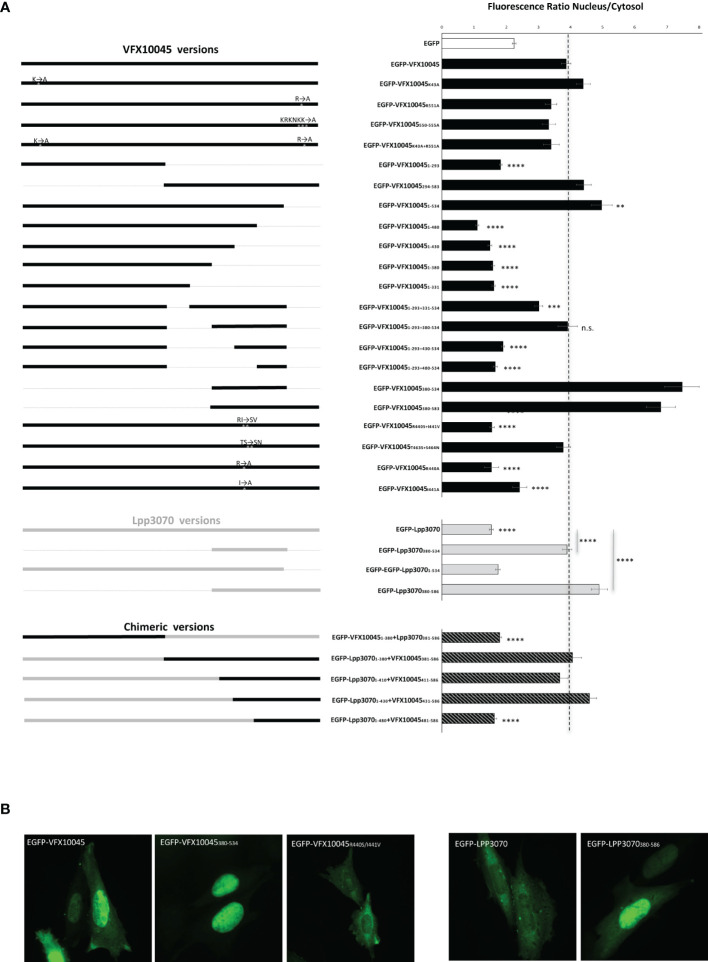
Effect of mutations on the subcellular localization of *L. pneumophila* effectors VFX10045 and LPP3070 in CHO cells. Plasmids encoding GFP fusions to different versions of *L. pneumophila* Icm/Dot substrates VFX10045 and Lpp3070 were used to transiently transfect CHO cells (representative images are shown in **Supplemental Figure S5**). **(A)** Graphical depiction of constructs (left; black bars, VFX10045 protein regions; grey bars, Lpp3070 protein regions; striped bars, chimeric proteins) and corresponding ratios of protein present in nucleus relatively to cytosol (right; Ratio Nucleus/Cytosol), calculated after quantification of the total GFP fluorescence in each of the two cell compartments in ImageJ. Bars, averages; error bars, standard error of the mean. For comparison a dashed line indicates the value obtained for full-length VFX10045. The results were obtained from the quantifications of three independent experiments, with a total of at least 60 cells analysed. Statistical analysis was made with student T-test by comparison with wild-type VFX10045 or with the indicated proteins; **, p<0.01; ***, p<0.001; ****, p<0.0001; n.s., non-significant. **(B)** Representative microscopy images of the indicated proteins.

To identify the mechanism responsible for the nuclear localization of VFX10045 (583 amino acid residues), we initially searched for NLS(s) using the softwares NLStradamus (Nguyen Ba et al., 2009), NLS MAPPER (Kosugi et al., 2009) and SeqNLS (Lin et al., 2012). Two highly scored putative canonical NLSs were identified that consisted of regions enriched in positively charged residues and containing the consensus sequence K-K/R-x-K/R between residues 42-45 (KKIK), and between residues 550-555 (KRKNKK) (**Figure S4**). To verify if these corresponded to the signal(s) directing the protein to the nuclear compartment, we performed site-directed mutagenesis on the plasmid encoding EGFP-VFX10045 followed by analyses of the localization of the mutant proteins in transfected CHO cells (**Figure 5** and **Figure S5**). In each case, to measure the impact of the engineered amino acid replacements on the nuclear targeting of EGFP-VFX10045, we calculated the amount of protein in the nucleus relatively to the protein in the cytosol. This was expressed as the ratio nucleus/cytosol, or N/C (see Materials and Methods for details), which in the case of EGFP-VFX10045 is approximately 4 (**Figure 5A**; representative microscopy images in **Figure S5** and immunoblots in **Figure S6**).

Nuclear localization of the mutant EGFP-VFX10045 proteins with single replacements K_43_A or R_551_A (expected to disrupt each putative NLSs), multiple replacements KRKNKK_550-555_AAAAAA or K_43_A combined with R_551_A was assessed, but no significant changes were observed relative to wild-type EGFP-VFX10045 (**Figure 5** and **Figure S5**). To search for non-canonical nuclear targeting region(s), we then examined the effect of deletions, starting by constructs containing only the N-terminal half of VFX10045 (residues M_1_-K_293_) or the C-terminal half region (residues I_294_-I_583_). Visualization of transfected cells showed that the C-terminal region (I_294_-I_583_) is essential and sufficient to target the protein to the nucleus, in contrast to the nonessential role of the N-terminal region (**Figure 5**). By analysing cells transfected with plasmids encoding increasingly larger sections from the C-terminus of VFX10045, we observed that a truncated EGFP-VFX10045_1-534_ retained the nuclear localization (**Figure 5A**) but smaller proteins were not localized in the nucleus (VFX10045_1-480_, VFX10045_1-430,_ VFX10045_1-380_ and VFX10045_1-331_). This excluded the last ~50 residues of VFX10045 from a relevant role in its nuclear targeting and attributed a fundamental role to the region between residues 294 and 534. Analysis of the localization in transfected cells of additional truncated EGFP-VFX10045 proteins, showed that the region between residues 380 and 534 has an essential role in the nuclear tropism of VFX10045 (**Figure 5**). In some of these experiments, to prevent unwanted diffusion to the nucleus because of a small protein size, we included in the constructs the N-terminal region (VFX10045_1-293_; **Figure 5A**), shown to be non-relevant for the nuclear targeting (see above).

### The Distinct Localization of Homologs VFX10045 and Lpp3070 Is Related to Two Amino Acid Differences

To help pinpointing the residues enabling nuclear targeting of VFX10045, we aimed to analyze the localization in transfected CHO cells of chimeras between VFX10045 and its 94% identical homolog Lpp3070, which is mostly cytosolic (**Figures S2, 3 **and** 4**). In initial experiments with truncated versions of Lpp3070 fused to EGFP we observed, as expected, that EGFP- Lpp3070_1-534_ does not localize to the nucleus but, surprisingly, that EGFP-Lpp3070_380-586_ or EGFP-Lpp3070_380-534_ concentrate in the nucleus (**Figure 5**, **Figures S5 **and** S6**). This suggests that the C-terminal region of Lpp3070 contains a cryptic nuclear targeting signal that is nonfunctional in the context of full-length Lpp3070. As regions between residues 380 and 584 of VFX10045 and Lpp3070 are 85% identical, the capacity of VFX10045 to migrate to the nucleus must be related to discrete differences in the nucleotide sequence of VFX10045. When we analyzed the localization of chimeras VFX10045-Lpp3070, this revealed that VFX10045_1-380_-Lpp3070_381-586_ did not localize to the nucleus, whereas Lpp3070_1-380_-VFX10045_381-583_ did (**Figures 5**, **S5 **and** S6**). This further confirmed that the C-terminal region of VFX10045 (from residue 381) can drive nuclear transport. Analysing the localization of additional chimeras with a shorter C-terminal of VFX10045 and longer portions of Lpp3070 (Lpp3070_1-410_-VFX_411-583_, Lpp3070_1-430_-VFX_431-583_ and Lpp3070_1-480_-VFX_481-583_) showed the relevance of residues 430 to 480 from VFX10045 in nuclear targeting (**Figure 5**). A comparison of the primary structure of the two proteins in this region (**Figure S4**) revealed two pairs of amino acids with non-conserved substitutions: R440 and I441 in VFX10045, corresponding to S443 and V444 in Lpp3070; and T463 and S464, corresponding to S466 and N467 in Lpp3070. Therefore, we performed additional site-directed mutagenesis on the plasmid encoding EGFP-VFX10045 generating vectors encoding EGFP-VFX10045_R440S+I441V_ and EGFP-VFX_T463S+S464N_ mutant proteins. While EGFP-VFX_T463S+S464N_ localized in the nucleus, the exchanges R440S and I440V abrogated transport of the mutant EGFP-VFX10045 protein to the nucleus, also observed in the corresponding 4HA tagged mutant during infection (**Figure 5B** and **Figure 4**). Subsequent independent replacements R440A and I441A confirmed and pinpointed the importance of both residues, particularly of R440, in the nuclear targeting of VFX10045 (**Figure 5** and **Figure S5**).

## Discussion

In this work, two novel Icm/Dot T4SS substrates from *L. pneumophila* were identified, firstly in the strain that caused one of the major worldwide outbreaks of Legionnaires’ disease, *L. pneumophila* PtVFX/2014 (effectors VFX05045 and VFX10045), and subsequently in clinically important strain *L. pneumophila* Paris (Lpp1450 and Lpp3070). As homologs VFX05045/Lpp1450 and VFX10045/Lpp3070 are transported into host cells it is likely that they exert an effector function to promote *L. pneumophila* infection. These two pairs of homologous effector proteins display an amino acid identity of >90%, but while VFX05045 and Lpp1450 show an identical and nonspecific localization in the host cell cytosol 24 hours post-infection, subcellular targeting of VFX10045 is different from Lpp3070. VFX10045 accumulates in the nucleus whereas Lpp3070 is mostly dispersed in the cell with sporadic patches, namely in regions of cell-cell contacts. The localization of VFX05045/Lpp1450 and VFX10045/Lpp3070 was mimicked by ectopic expression of the GFP tagged effectors in CHO cells. The localization of VFX10045 prompted us to search for signals that would convey this nucleotropism to VFX10045 but not to homolog Lpp3070. These studies compared the subcellular localization of VFX10045 and Lpp3070 variants obtained by site-directed mutagenesis, consisting of truncations, single amino acid substitutions or chimeric construct containing swapped larger protein regions. Taking all the results together, we found that region VFX1004_380-530_ is necessary and sufficient for transport of EGFP to the nucleus and assigned to specific amino acid residues Arg440 and Ile441 an essential role in this process. The region comprising these residues does not contain the classical motifs K-K/R-X-K/R or R/K-X_10-12-_K-R-X-K, respectively for monopartite and bipartite NLSs (Nguyen Ba et al., 2009). This suggests that VFX10045 may use a non-classical NLS or employ a mechanism for nuclear translocation which does not involve direct binding to importin-α, such as piggybacking on an NLS containing protein or using a new mechanism of nucleocytoplasmic transport. The fact that the truncated variant Lpp3070_380-530_ is also nuclear bound also suggests a presence of a cryptic NLS therein, which is inactive in the context of a full-length Lpp3070 protein.

In previous studies, Kaneko and coworkers (Kaneko et al., 2018) had tested the translocation of Lpp3070 *via* the Icm/Dot T4SS by means of protein fusions to CyaA. Using this methodology, they were not able to confirm delivery of Lpp3070 to the cytosol of macrophages during infection. However, this is not unprecedented, as previously reported cases have shown that translocation assays relying on Cya detection method may yield false negative results (de Felipe et al., 2008). In this study, they identified two predicted Src homology 2 (SH2) domains between amino acid residues 281-370 and 443-532 (corresponding to residues 281-370 and 440-529 in VFX10045; Figure S4). SH2 domains bind phosphorylated tyrosine (pTyr) and are fundamental in signal transduction in mammalian cells, namely in receptor tyrosine kinase pathways. In this groundbreaking study, the authors identified SH2 domains in *L. pneumophila* effector proteins that are distinct in specificity and sequence from their mammalian counterparts, anticipating the existence of pTyr-superbinding characteristics in bacterial effectors that could be key in bacterial-host interactions. Interestingly, key residues Arg440 and Ile441 in VFX10045 are contained within the second predicted SH2 motif, which may hypothesize a role in conferring different specificities and/or affinities between SH2 domains of the two homologs VFX10045 and Lpp3070, and consequently on their function during infection. Thus far, the function of SH2 domain containing proteins from *L. pneumophila* remains unknown (Kaneko et al., 2018), although a possible role in signaling may be foreseen.

A documented example of a strain-specific functional divergence between *L. pneumophila* effector homologs is the case of *L. pneumophila* Paris RomA versus *L. pneumophila* Lp02 LegAS4. Both nucleomodulins act as histone methyltransferases but catalyse methylation on different lysines on histone H3 (K14 or K4, respectively), yielding distinct outcomes in terms of target genes and downstream transcription outcomes. As put forth by Price and Kwaik (Price and Kwaik, 2013), these differences may be caused by a stretch of 13 amino acids present only at the N-terminal of LegAS4, by a low similarity region starting at residue 66 (80 in LegAS4) or by small differences in their NLSs that could be targeting the effectors to different sites within the nucleus. A second example is the functional and localization difference found in AnkB from strains *L. pneumophila* Paris and 130b. *L. pneumophila* Paris AnkB lacks the last 18 residues present in *L. pneumophila* 130b AnkB which are responsible for the anchoring of the latter to the LCV membrane (Price et al., 2010; Perpich et al., 2017), which may assign different subcellular localizations and functions to this effector. Thus, the differences in function of homologous effectors belonging to distinct *L. pneumophila* strains, such as the ones observed in RomA/LetAS4 and AnkB, and anticipated for VFX10045/Lpp3070, highlight the necessity of independent analyses even in highly identical effectors, and support their evolutionary divergence towards the acquisition of distinct functions.

## Data Availability Statement

The datasets presented in this study can be found in online repositories. The names of the repository/repositories and accession number(s) can be found in the article/**Supplementary Material**.

## Author Contributions

IM, SS, PG and IF performed the experiments. VB, JG and IF did the bioinformatics analyses. VB, JG, LM and IF conceived and designed the project and interpreted the data. VB, LM and IF wrote the manuscript. All authors contributed to the article and approved the submitted version.

## Funding

This project has been funded by: Research Grant 2016 by the European Society of Clinical Microbiology and lnfectious Diseases (ESCMID) to IF; by National funds from FCT - Fundação para a Ciência e a Tecnologia, I.P., in the scope of the project UIDP/04378/2020 and UIDB/04378/2020 of the Research Unit on Applied Molecular Biosciences - UCIBIO and the project LA/P/0140/2020 of the Associate Laboratory Institute for Health and Bioeconomy - i4HB.

## Conflict of Interest

The authors declare that the research was conducted in the absence of any commercial or financial relationships that could be construed as a potential conflict of interest.

## Publisher’s Note

All claims expressed in this article are solely those of the authors and do not necessarily represent those of their affiliated organizations, or those of the publisher, the editors and the reviewers. Any product that may be evaluated in this article, or claim that may be made by its manufacturer, is not guaranteed or endorsed by the publisher.
