# Supplementary material for: A Search for Novel Legionella pneumophila Effector Proteins Reveals a Strain Specific Nucleotropic Effector
Source: Front Cell Infect Microbiol. 2022 May 31;12:864626. doi: 10.3389/fcimb.2022.864626 (PMC9195298; doi:10.3389/fcimb.2022.864626)
Supplement: Supplementary file 1 [file DataSheet_1.pdf]

Supplemental FIGURE S1

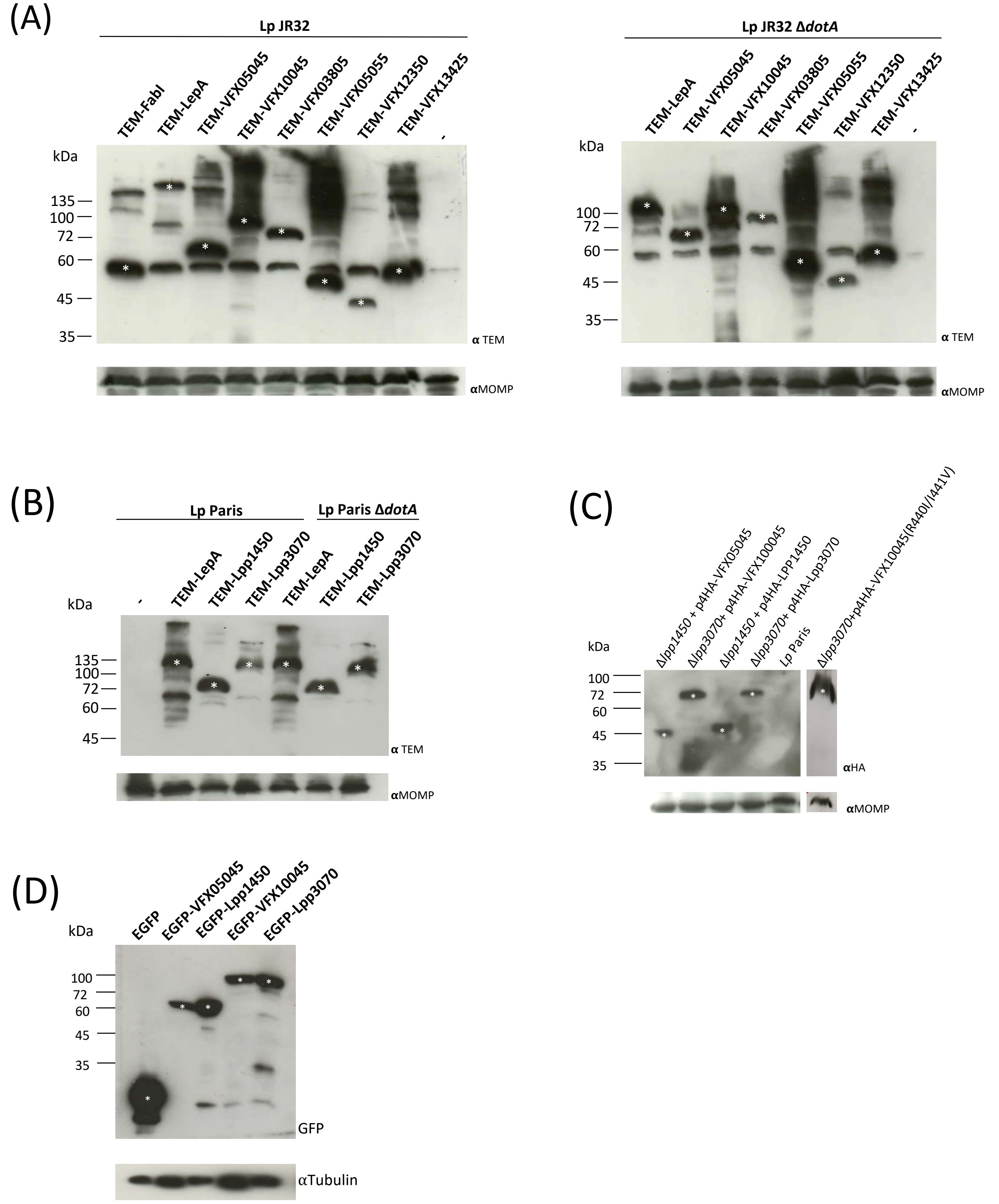

Supplemental FIGURE S1. Immunoblotings of *L. pneumophila* Icm/Dot substrate proteins identified in this work, fused to TEM-1 β-lactamase (A and B), HA epitope (C) or EGFP (D). (**A-C)** Crude extracts of the *L. pneumophila* strains were prepared after growth in the presence of IPTG and proteins separated on SDS-PAGE. Immunoblots were carried out using primary antibodies against TEM-1 β-lactamase (mouse), HA epitope (rat) or *L. pneumophila* MOMP (rabbit) and appropriate secondary anti-HRP. (**D)** Western blot with extracts from cells transfected as in Fig. 3. Primary antibodies against GFP (goat) or α-tubulin (mouse) were used followed by corresponding secondary HRP coupled antibodies.

Supplemental Figure S2

	Lp Paris Lpp1450	Lp Lens Lpl2045	Lp Corby Lpc1557	Lp Alcoy Lpa03013
Lp Pt/VFX2014 VFX05045	90%	88% (aa 19-351)	67% (aa 17-353)	67% (aa 17-353)
Lp Paris Lpp1450	-	87%	67%	67%
Lp Lens Lpl2045	-	-	65%	66%
Lp Corby Lpc1557	-	-	-	94%

	Lp Paris Lpp3070	Lp Corby Lpc3314	Lp Alcoy Lpa04392
Lp Pt/VFX2014 VFX10045	94%	93%	94%
Lp Paris Lpp3070	-	98%	98%
Lp Corby Lpc3314	-	-	99%

Supplemental FIGURE S2. Degrees of identity at the amino acid level of VFX05045 and VFX10045 homologs in other *L. pneumophila* strains.

Supplemental FIGURE S3

(A)

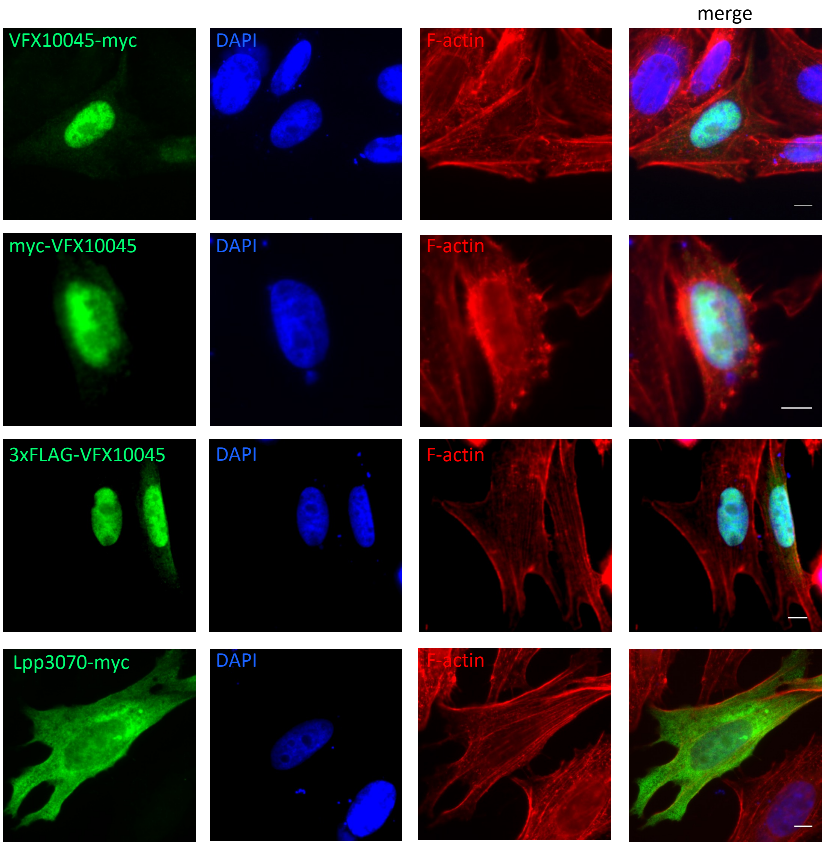

(B)

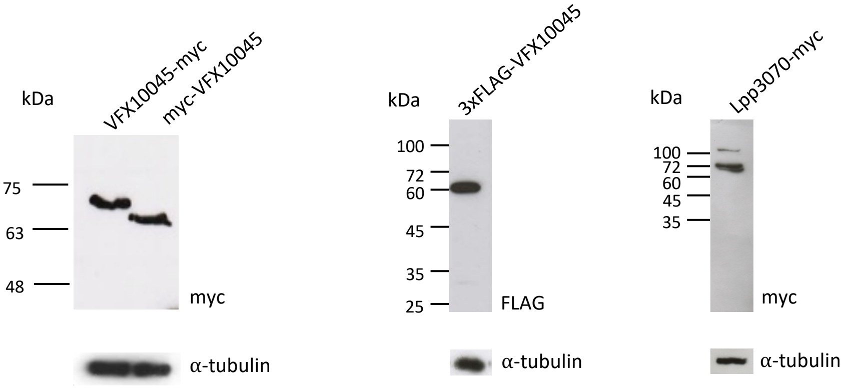

Supplemental FIGURE S3. Subcellular localization in CHO cells of VFX10045 and Lpp3070 fused to different epitopes. Plasmid pEF6a derivatives encoding myc-VFX10045, VFX10045-myc, 3xFLAG-VFX10045 and Lpp3070-myc were used to transiently transfect CHO mammalian cells. (**A**) Cells were fixed with 4% PFA (w/v), permeabilized with 0.1% Triton X100 (v/v) and labeled with Phalloidin-AlexaFluor-555 and DAPI. Scale bar, 5 µm. (**B**) Immunoblotting of the corresponding cell extracts with anti-myc, anti-FLAG or anti-α-tubulin primary antibodies and secondary HRP-coupled antibodies.

Supplemental FIGURE S4

```
                            1                                30                             60
VFX10045(Lp VFX)     MTIECYSIDGDECILRKEYDDAPDSEKFSGKAVIYTNLPLIKKIKREIAKNKLQPIIVFNGSSRQSMFMEQLNALREKKLFRGSFCLALQ 90
Lpp3070 (Lp Paris)   MTIECYSIDGDECILRKEYDDAPDSEKFSGKAVIYTNLPLIKKIKHEIAKNKLHPIIVFNGSSRQSMFMEQLNALREKKLFRGSFCLALQ 90
Lpa04394(Lp Alcoy)   MTIECYSIDGDECILRKEYDDAPDSEKFSGKAVIYTNLPLIKKIKHEIAKNKLHPIIVFNGSSRQSMFMEQLNALREKKLFRGSFCLALQ 90
Lpc3314 (Lp Corby)   MTIECYSIDGDECILRKEYDDAPDSEKFSGKAVIYTNLPLIKKIKHEIAKNKLHPIIVFNGSSRQSMFMEQLNALREKKLFRGSFCLALQ 90
                     *********************************************:*******:************************************

                                                     120                            150
VFX10045(Lp VFX)     RLCDYLKVELDKTLLPDITGDLEYGTTFNRIMDEVLNIEWLDNKKLQHKHVPYPDYNENWHAKLDPFKRILLFAQIQKIRNDHPDEDIIF 180
Lpp3070 (Lp Paris)   KLCDYLKVELDKTLLPDITGDLEYGTTFNRIMDEVLNIEWLDNKKLQHKHVPYPDYNENWHAKLDPFKRILLFAQIQKIRNDHPDEDIIF 180
Lpa04394(Lp Alcoy)   KLCDYLKVELDKTLLPDITGDLEYGTTFNRIMDEVLNIEWLDNKKLQHKHVPYPDYNENWHAKLDPFKRILLFAQIQKIRNDHPDEDIIF 180
Lpc3314 (Lp Corby)   KLCDYLKVELDKTLLPDITGDLEYGTTFNRIMDEVLNIEWLDNKKLQHKHVPYPDYNENWHAKLDPFKRILLFAQIQKIRNDHPDEDIIF 180
                     :*****************************************************************************************

                                                     210                            240
VFX10045(Lp VFX)     NFIDDKDDILSTLKKYFINYSYMIPHGVTLRLNHYDGSNVSSYASIKGTGRPYKNYRQIVIDMHEHVYNLDAFAETINQNDKFSEIPAVR 270
Lpp3070 (Lp Paris)   NFIDDKDDILSTLKKYFINYSYMIPHGVTLRLNHYDGSNVSSYASIKGTGRPYKNYRQIVKDMHEHVYNLDAFAETINQNDKFSEIPAVR 270
Lpa04394(Lp Alcoy)   NFIDDKDDILSTLKKYFINYSYMIPHGVTLRLNHYDGSNVSSYASIKGTGRPYKNYRQIVKDMHEHVYNLDAFAETINQNDKFSEIPAVR 270
Lpc3314 (Lp Corby)   NFIDDKDDILSTLKKYFINYSYMIPHGVTLRLNHYDGSNVSSYASIKGTGRPYKNYRQIVKDMHEHVYNLDAFAETINQNDKFSEIPAVR 270
                     ******************************************************************************************

                                                     300          SH2               330
VFX10045(Lp VFX)     QILEHEFFVSDPDDVVSCLDNGKIPFVIRKSKSQISGYIMFTAVYKTSQGIFANRYGINTEGKLYKFFDSQIEKMDIDSEGIIAALEREI 360
Lpp3070 (Lp Paris)   QILEHEFFVSDPDDVVSCLDKGKIPFVIRKSKSQISGYIMFTAVYKTSQGIFANRYGINTEGELYKFFDSQIEKMDIDSEGIIAALEREI 360
Lpa04394(Lp Alcoy)   QILEHEFFVSDPDDVVSCLDKGKIPFVIRKSKSQISGYIMFTAAYKTSQGIFANRYGINTEGELYKFFDSQIEKMDIDSEGIIAALEREI 360
Lpc3314 (Lp Corby)   QILEHEFFVSDPDDVVSCLDKGKIPFVIRKSKSQISGYIMFTAAYKTSQGIFANRYGINTEGELYKFFDSQIEKMDIDSEGIIAALEREI 360
                     ********************:**********************.******************:***************************

                                                     390                            420
VFX10045(Lp VFX)     VITKMIIEKTENLSKIPWKSQPEKKIIDLDYQENGEKEAETNPSELFTPMTN---SFHSSDRRENKQKSLCSQLRSCKFFVSRINNAESE 447
Lpp3070 (Lp Paris)   VITKMIIEKTENLSKIPWKSQPEKKNTDLDYQENGEKEAETNPSELFTPMTKKTKSPHSSDRRENKQKSLCSQLRSCKFFVSSVNNAESE 450
Lpa04394(Lp Alcoy)   VITKMIIEKTENLSKIPWKSQPEKKNNDLDYQENGEKEAETNPSELFTPMTKKTKSPQ----RENKQKSLCSQLRSCKFFVSSVNNAESE 446
Lpc3314 (Lp Corby)   VITKMIIEKTENLSKIPWKSQPEKKNNDLDYQENGEKEAETNPSELFTPMTKKTKSPQ----RENKQKSLCSQLRSCKFFVSSVNNAESE 446
                     *************************  ************************:   * :    ******************** :******

                                                          480     SH2                     510
VFX10045(Lp VFX)     LNKKQLPFLIRPSRATSNEYYMFTTVCKTSKGIFNHRYGIDRKGNLYSFDKDGTQKVNIMPKGIIATLKKEIVEQTQKIEKAQDVAQITW 537
Lpp3070 (Lp Paris)   LNKKQLPFLIRPSRASNNEYYLFTTVCKTSKGIFNHRYGIDRKGNLYSFDKDGTQKVNIMPKGIIATLKKEIAEETEKIEKVKDIAQITW 540
Lpa04394(Lp Alcoy)   LNKKQLPFLIRPSRATSNEYYMFTTVCKTSKGIFNHRYGIDRKGNLYSFDKDGTQKVNIMPKGIIATLKKEIAEETEKIEKVKDIAQITW 536
Lpc3314 (Lp Corby)   LNKKQLPFLIRPSRATSNEYYMFTTVCKTSKGIFNHRYGIYRKGNLYSFDKDGTQKVNIMPKGIIATLKKEIAEETEKIEKVKDIAQITW 536
                     ***************:.****:**************************************************.*:*:****.:*:*****

                                                          570
VFX10045(Lp VFX)     IPEEDKSPNSSTKRKNKKPEWHLKFFNENGDSTQFSEITPTPCKNI   583
Lpp3070 (Lp Paris)   VPDEYKSSKSSIKGKNRKPEWHLKFFNENGDSAQVSNTTPTLCKNI   586
Lpa04394(Lp Alcoy)   VPDEYKSSKSSIKGKNRKPEWHLKFFNENGDSAQVSNTTPTLCKNI   582
Lpc3314 (Lp Corby)   VPDEYKSSKSSIKGKNRKPEWHLKFFNENGDSAQVSNTTPTLCKNI   582
                     :*:* ** :** * **:***************:*.*: *** ****
```

Supplemental Figure S4. Alignment of VFX10045 and homologs from strains *L. pneumophila* Paris, Alcoy and Corby. Alignment was performed with Clustal Omega and symbols below the sequences indicate amino acid residues that are conserved (*), with strong similarity (:) or weak similarity (.). Putative NLSs identified *in silico* (see text) are highlighted in yellow. Amino acids substituted in this work are in bold, with the two residues fundamental for VFX10045 nuclear localization highlighted in green (Arg440 and Ile441). The two putative SH2 (Src homology 2) domains identified in Lpp3070 (Kaneko *et al.*, 2018) are depicted (blue bars).

Supplemental Figure S5

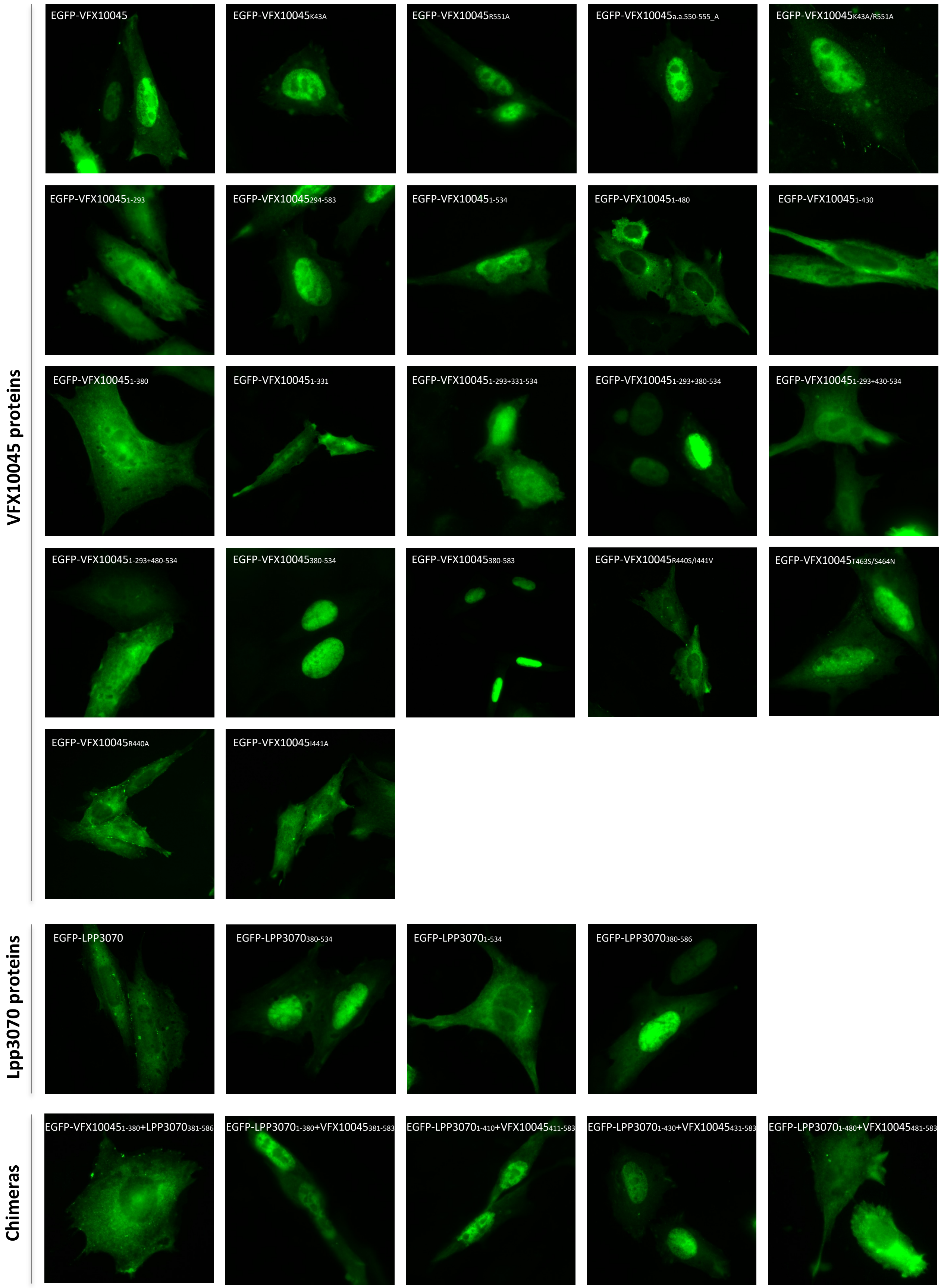

Supplemental Figure S5. Effect of mutations in the subcellular localization of *L. pneumophila* effectors VFX10045 and LPP3070 in CHO cells. Plasmids encoding GFP fusions to different versions of *L. pneumophila* Icm/Dot substrates VFX10045 and Lpp3070 were used to transiently transfect CHO cells. Cells were fixed with 4% PFA (w/v). Representative images are shown.

Supplemental Figure S6

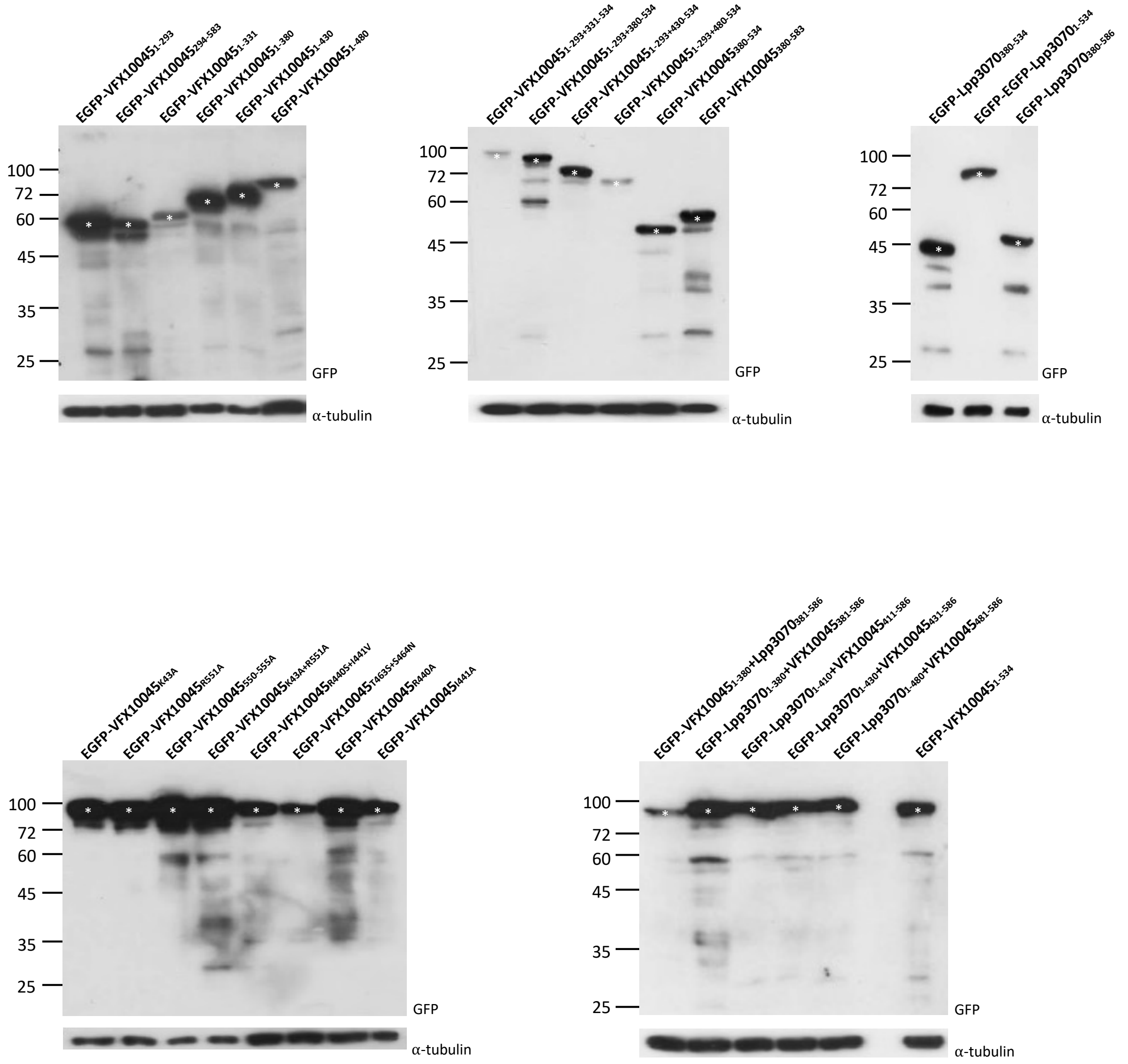

Supplemental Figure S6. Immunoblotting of extracts from cells transfected as in Fig. S4. Primary antibodies against GFP (goat) or α-tubulin (mouse) were used followed by corresponding HRP coupled secondary antibodies.
