# Supplementary material for: A Search for Novel Legionella pneumophila Effector Proteins Reveals a Strain Specific Nucleotropic Effector
Source: Front Cell Infect Microbiol. 2022 May 31;12:864626. doi: 10.3389/fcimb.2022.864626 (PMC9195298; doi:10.3389/fcimb.2022.864626)
Supplement: Supplementary file 2 [file DataSheet_2.pdf]

TABLE S1. *Legionella pneumophila* (*Lp*) strains used in this work.

Name	Relevant genotype/Construction	Reference/Source
L. pneumophila Pt/VFX2014	Wild-type	Borges V *et al*., 2016
L. pneumophila JR32	*Lp* Philadelphia-1, SmR, r-m+	Sadosky AB *et al.*, 1993
L. pneumophila LELA3118	*Lp* JR32 dotA::Tn903dlllacz3118	Sadosky AB *et al.*, 1993
L. pneumophila Paris	Wild-type	X. Charpentier
L. pneumophila Paris dotA	Icm/Dot$^-$	X. Charpentier
LPIF131	*Lp* JR32 + pXDC61-FabI (TEM-FabI)	This work
LPIF134	*Lp* JR32 + pXDC61-LepA (TEM-LepA)	This work
LPIF135	*Lp* JR32 *dotA* + pXDC61-LepA (Icm/Dot$^-$ TEM-LepA)	This work
LPIF166	*Lp* JR32 + pIF387 (TEM-VFX03805)	This work
LPIF167	*Lp* JR32 + pIF388 (TEM-VFX05045)	This work
LPIF168	*Lp* JR32 + pIF389 (TEM-VFX05055)	This work
LPIF170	*Lp* JR32 + pIF391 (TEM-VFX12350)	This work
LPIF171	*Lp* JR32 + pIF392 (TEM-VFX13425)	This work
LPIF173	*Lp* JR32 + pIF395 (TEM-VFX09510)	This work
LPIF174	*Lp* JR32 + pIF396 (TEM-VFX06065)	This work
LPIF175	*Lp* JR32 + pIF397 (TEM-VFX10045)	This work
LPIF178	*Lp* JR32 *dotA* + pIF387 (Icm/Dot$^-$ TEM-VFX03805)	This work
LPIF179	*Lp* JR32 *dotA* + pIF388 (Icm/Dot$^-$ TEM-VFX05045)	This work
LPIF180	*Lp* JR32 *dotA* + pIF389 (Icm/Dot$^-$ TEM-VFX05055)	This work
LPIF182	*Lp* JR32 *dotA* + pIF391 (Icm/Dot$^-$ TEM-VFX12350)	This work
LPIF183	*Lp* JR32 *dotA* + pIF392 (Icm/Dot$^-$ TEM-VFX13425)	This work
LPIF185	*Lp* JR32 *dotA* + pIF395 (Icm/Dot$^-$ TEM-VFX09510)	This work
LPIF186	*Lp* JR32 *dotA* + pIF396 (Icm/Dot$^-$ TEM-VFX06065)	This work
LPIF187	*Lp* JR32 *dotA* + pIF397 (Icm/Dot$^-$ TEM-VFX10045)	This work
LPIF221	*Lp* Paris + pIF435 (TEM-Lpp1450)	This work
LPIF222	*Lp* Paris + pIF436 (TEM-Lpp3070)	This work
LPIF223	*Lp* Paris + pXDC61-LepA (TEM-LepA)	This work
LPIF225	*Lp* Paris *dotA* + pIF435 (Icm/Dot$^-$ TEM-Lpp1450)	This work
LPIF226	*Lp* Paris *dotA* + pIF436 (Icm/Dot$^-$ TEM-Lpp3070)	This work
LPIF227	*Lp* Paris *dotA* + pXDC61-LepA (Icm/Dot$^-$ TEM-LepA)	This work
LPIF234	*Lp* Paris Δ*lpp3070*::kan^r	This work
LPIF235	*Lp* Paris Δ*lpp1450*::kan^r	This work
LPIF246	*Lp* Paris Δ*lpp3070*::kan^r + pIF479 (4HA-VFX10045)	This work
LPIF249	*Lp* Paris Δ*lpp3070*::kan^r + pIF478 (4HA-Lpp3070)	This work
LPIF252	*Lp* Paris Δ*lpp1450*::kan^r + pIF477 (4HA-VFX05045)	This work
LPIF255	*Lp* Paris Δ*lpp1450*::kan^r + pIF480 (4HA-Lpp1450)	This work
LPIF261	*Lp* Paris Δ*lpp3070*::kan^r + pIF487 (4HA-VFX10045$_{R440I+S441V}$)	This work
LPIF263	*Lp* Pt/VFX2014 Δ*vfx10045*::kan^r	This work

TABLE S2. Plasmids used in this work.

Plasmid	Fusion protein	Construction	Reference
pEGFP-c1 derivatives (EGFP fusion proteins)			
pEGFP-c1		Mammalian expression vector for N-terminal EGFP fusion proteins	Clontech
pIM1	EGFP-VFX05045	Insert obtained by PCR amplification of *vfx05045* using oligos 1940 and 1941 and template chromosomal DNA from strain *L. pneumophila* Pt/VFX2014, followed by digestion with *Bam*HI and *Hind*III and insertion at pEGFP-c1 *Bgl*II-*Hind*III	This work
pIM2	EGFP-VFX10045	Insert obtained by PCR amplification of *vfx100045* using oligos 1952 and 1953 and template chromosomal DNA from strain *L. pneumophila* Pt/VFX2014, followed by digestion with *Bam*HI and *Kpn*I and insertion at pEGFP-c1	This work
pGF1	EGFP-VFX10045$_{K43A}$	Site-directed mutagenesis of *vfx10045* by PCR with oligos 2353 and 2354 using as template DNA pIM2.	This work
pIF418	EGFP-VFX10045$_{R551A}$	Site-directed mutagenesis of *vfx10045* by PCR with oligos 2355 and 2356 using as template DNA pIM2.	This work
pIF419	EGFP-VFX10045$_{K43A+R551A}$	Site-directed mutagenesis of *vfx10045* by PCR with oligos 2355 and 2356 using as template DNA pGF1.	This work
pIF420	EGFP-VFX10045$_{294\text{-}583}$	Site-directed mutagenesis of *vfx10045* by PCR with oligos 2400 and 1952 using as template pIM2, followed by digestion with *Bam*HI and *Kpn*I and insertion at pEGFP-c1.	This work
pIF421	EGFP-VFX10045$_{1\text{-}293}$	Site-directed mutagenesis of *vfx10045* by PCR with oligos 2401 and 1953 using as template pIM2, followed by digestion with *Bam*HI and *Kpn*I and insertion at pEGFP-c1.	This work
pIF422	EGFP-VFX10045$_{550\text{-}555::Ala}$	Site-directed mutagenesis of *vfx10045* (KRKNKK->AAAAAA) by PCR with oligos 2423 and 2424 using as template DNA pIF418.	This work
pIF423	EGFP-VFX10045$_{1\text{-}331}$	Site-directed mutagenesis by PCR of *vfx10045* with oligos 1953 and 2426 using as template pIM2, followed by digestion with *Bam*HI and *Kpn*I and insertion at pEGFP-c1.	This work
pIF424	EGFP-VFX10045$_{1\text{-}380}$	Truncation of VFX10045 was accomplished by PCR with oligos 1953 and 2427 using as template DNA pIM2, followed by digestion with *Bam*HI and *Kpn*I and insertion at pEGFP-c1	This work
pIF425	EGFP-VFX10045$_{1\text{-}430}$	Truncation of VFX10045 was accomplished by PCR with oligos 1953 and 2428 using as template DNA pIM2, followed by digestion with *Bam*HI and *Kpn*I and insertion at pEGFP-c1	This work
pIF426	EGFP-VFX10045$_{1\text{-}480}$	Truncation of VFX10045 was accomplished by PCR with oligos 1953 and 2429 using as template DNA pIM2, followed by digestion with *Bam*HI and *Kpn*I and insertion at pEGFP-c1	This work
pIF427	EGFP-VFX10045$_{1\text{-}534}$	Truncation of VFX10045 was accomplished by PCR with oligos 1953 and 2430 using as template DNA pIM2 followed by digestion with *Bam*HI and *Kpn*I and insertion at pEGFP-c1	This work
pIF430	EGFP-VFX10045$_{1\text{-}293+480\text{-}534}$	Insert obtained from overlap PCR of *vfx10045* (PCR1 with oligos 1953 and 2456, PCR2 with oligos 2457 and 2430) digested with *Kpn*I and *Bam*HI and inserted at pEGFP-c1	This work
pIF431	EGFP-VFX10045$_{1\text{-}293+430\text{-}534}$	Insert obtained from PCR of *vfx10045* using oligos 2484 and 2430 followed by digestion with *Xho*I and *Bam*HI and insertion at pIF421	This work
pIF432	EGFP-VFX10045$_{1\text{-}293+380\text{-}534}$	Insert obtained from PCR of *vfx10045* using oligos 2485 and 2430 followed by digestion with *Xho*I and *Bam*HI and insertion at pIF421	This work
pIF433	EGFP-VFX10045$_{1\text{-}293+331\text{-}534}$	Insert obtained from PCR of *vfx10045* using oligos 2486 and 2430 followed by digestion with *Xho*I and *Bam*HI and insertion at pIF421	This work
pIF434	EGFP-VFX10045$_{380\text{-}534}$	Insert obtained from PCR of *vfx10045* using oligos 2495 and 2430 followed by digestion with *Kpn*I and *Bam*HI and insertion at pEGFP-c1	This work
pIF437	EGFP-Lpp1450	Insert obtained from PCR of *lpp1450* using oligos 2445 and 2446 followed by digestion with *Kpn*I and *Xba*I and insertion at pEGFP-c1	This work
pIF438	EGFP-Lpp3070	Insert obtained from PCR of *lpp3070* using oligos 1953 and 2447 followed by digestion with *Kpn*I and *Xba*I and insertion at pEGFP-c1	This work
pIF440	EGFP-Lpp3070$_{380\text{-}534}$	Insert obtained from PCR of *lpp3070* using oligos 2534 and 2535 followed by digestion with *Kpn*I and *Bam*HI and insertion at pEGFP-c1	This work
pIF443	EGFP-Lpp3070$_{380\text{-}586}$	Insert obtained from PCR of *lpp3070* using oligos 2534 and 2447 followed by digestion with *Kpn*I and *Xba*I and insertion at pEGFP-c1	This work
pIF444	EFGP-VFX10045$_{380\text{-}583}$	Insert obtained from PCR of *vfx10045* using oligos 2495 and 1953 followed by digestion with *Kpn*I and *Bam*HI and insertion at pEGFP-c1	This work
pIF445	EGFP-Lpp3070$_{1\text{-}534}$	Insert obtained from PCR of *lpp3070* using oligos 1953 and 2535 followed by digestion with *Kpn*I and *Xba*I and insertion at pEGFP-c1	This work
pIF448	EFGP-VFX10045$_{1\text{-}380}$-LPP3070$_{381\text{-}586}$	Insert obtained from PCR of *lpp3070* using oligos 2534 and 2447 followed by digestion with *Kpn*I and *Xba*I and insertion at pIF424	This work
pIF449	EFGP-LPP3070$_{1\text{-}380}$-VFX10045$_{381\text{-}586}$	Insert obtained from PCR of *lpp3070* using oligos 2552 and 2553 followed by digestion with PstI and *Kpn*I and insertion at pIF444	This work
pIF468	EFGP-LPP3070$_{1\text{-}410}$-VFX10045$_{410\text{-}583}$	Insert obtained from overlap PCR (PCR1 of *lpp3070* with oligos 1953 and 2739, PCR2 of *vfx1004*5 with oligos 2738 and 1952) digested with *Kpn*I and *Bam*HI and inserted at pEGFP-c1	This work
pIF471	EFGP-LPP3070$_{1\text{-}430}$-VFX10045$_{431\text{-}586}$	Insert obtained from overlap PCR (PCR1 of *lpp3070* with oligos 1953 and 2774, PCR2 of *vfx1004*5 with oligos 2791 and 1952) digested with *Kpn*I and *Bam*HI and inserted at pEGFP-c1	This work
pIF473	EFGP-LPP3070$_{1\text{-}480}$-VFX10045$_{481\text{-}586}$	Insert obtained from overlap PCR (PCR1 of *lpp3070* with oligos 1953 and 2774, PCR2 of *vfx1004*5 with oligos 2790 and 1952) digested with *Kpn*I and *Bam*HI and inserted at pEGFP-c1	This work
pIF475	EGFP-VFX10045$_{R440S+I441V}$	Insert obtained from overlap PCR of *vfx10045* (PCR1 with oligos 1953 and 2810, PCR2 with oligos 2809 and 1952) digested with *Kpn*I and *Bam*HI and inserted at pEGFP-c1	This work
pIF476	EGFP-VFX10045$_{T463S+S464N}$	Insert obtained from overlap PCR of *vfx10045* (PCR1 with oligos 1953 and 2812, PCR2 with oligos 2811 and 1952) digested with *Kpn*I and *Bam*HI and inserted at pEGFP-c1	This work
pIF481	EGFP-VFX10045$_{R440A}$	Insert obtained from overlap PCR of *vfx10045* (PCR1 with oligos 1953 and 2830, PCR2 with oligos 2829 and 1952) digested with *Kpn*I and *Bam*HI and inserted at pEGFP-c1	This work
pIF483	EGFP-VFX10045$_{I441A}$	Insert obtained from overlap PCR of *vfx10045* (PCR1 with oligos 1953 and 2832, PCR2 with oligos 2835 and 1952) digested with *Kpn*I and *Bam*HI and inserted at pEGFP-c1	This work
pEF6a derivatives (myc fusion proteins)			
pEF6a		Expression vector for myc fusion proteins in mammalian cell lines	Invitrogen
pIF442	3xFLAG-VFX10045	Insert obtained by PCR amplification of 3xFLAG-*vfx10045* (in plasmid pGT2, unpublished) using oligos 2533 and 1952 followed by digestion with *Kpn*I and *EcoR*I and insertion at pEF6a	
pIF454	VFX10045-myc	Insert obtained by PCR amplification of *vfx10045* using oligos 2623 and 1952 followed by digestion with *Bam*HI and *Not*I and insertion at pEF6a	This work
pIF455	myc-VFX10045	Insert obtained by PCR amplification of *vfx10045* using oligos 2602 and 1952 followed by digestion with *Bam*HI and *Kpn*I and insertion at pEF6a	This work
pIF488	Lpp3070-myc	Insert obtained by PCR amplification of *lpp3070* using oligos 2622 and 2852 followed by digestion with *Bam*HI and *Not*I and insertion at pEF6a	This work

pXDC61 derivatives (TEM-1 fusion proteins)			
pXDC61		Vector for expression of N-terminal TEM-1 β-lactamase fusions in *L. pneumophila*	Charpentier et al., 2009
pIF387	TEM-VFX03805	Insert obtained by PCR amplification of *vfx03805* using oligos 1938 and 1939 followed by digestion with *Bam*HI and *Hind*III and insertion at pXDC61	This work
pIF388	TEM-VFX05045	Insert obtained by PCR amplification of *vfx05045* using oligos 1940 and 1941 followed by digestion with *Bam*HI and *Hind*III and insertion at pXDC61	This work
pIF389	TEM-VFX05055	Insert obtained by PCR amplification of *vfx05045* using oligos 1944 and 1945 followed by digestion with *Bam*HI and *Hind*III and insertion at pXDC61	This work
pIF391	TEM-VFX12350	Insert obtained by PCR amplification of *vfx05045* using oligos 1954 and 1955 followed by digestion with *Bam*HI and *Hind*III and insertion at pXDC61	This work
pIF392	TEM-VFX13425	Insert obtained by PCR amplification of *vfx05045* using oligos 1956 and 1957 followed by digestion with *Bam*HI and *Hind*III and insertion at pXDC61	This work
pIF395	TEM-VFX09510	Insert obtained by PCR amplification of *vfx09510* using oligos 1950 and 1951 followed by digestion with *Bam*HI and *Hind*III and insertion at pXDC61	This work
pIF396	TEM-VFX06065	Insert obtained by PCR amplification of *vfx06045* using oligos 1948 and 1949 followed by digestion with *Bam*HI and *Kpn*I and insertion at pXDC61	This work
pIF397	TEM-VFX10045	Insert obtained by PCR amplification of *vfx10045* using oligos 1952 and 1953 followed by digestion with *Bam*HI and *Kpn*I and insertion at pXDC61	This work
pIF435	TEM-LPP1450	Insert obtained by PCR amplification of *lpp1450* using oligos 2445 and 2446 followed by digestion with *Kpn*I and *Xba*I and insertion at pXDC61	This work
pIF436	TEM-LPP3070	Insert obtained by PCR amplification of *lpp3070* using oligos 1953 and 2447 followed by digestion with *Kpn*I and XbaI and insertion at pXDC61	This work
pMMB207c derivatives (4HA fusion proteins)			
pIF376	Ptac-4HA	Insert obtained by annealing of oligos 1822 and 1823, yielding DNA fragment with EcoRI and BamHI cohesive ends, and substitution of TEM at same sites of pXDC61	This work
pIF477	4HA-VFX05045	Insert obtained by PCR amplification of *vfx05045* using oligos 1940 and 1942, followed by digestion with BamHI and HindIII and insertion at pIF376	This work
pIF478	4HA-Lpp3070	Insert obtained by PCR amplification of *lpp3070* using oligos 2604 and 2447, followed by digestion with BamHI *and* XbaI and insertion at pIF376	This work
pIF479	4HA-VFX10045	Insert obtained by PCR amplification of *vfx10045* using oligos 2604 and 1952, followed by digestion with BamHI and insertion at pIF376	This work
pIF480	4HA-Lpp1450	Insert obtained by PCR amplification of *lpp1450* using oligos 2737 and 2446, followed by digestion with XbaI and insertion at pIF376	This work
pIF487	4HA-VFX10045$_{R440S+I441V}$	Insert obtained from overlap PCR of *vfx10045* (PCR1 with oligos 2604 and 2810, PCR2 with oligos 2809 and 2850) digested with *Bam*HI and inserted at pIF376	This work
pUC18 derivatives (for gene replacement with kan^R in L. pneumophila)			
pIF463	pUC18::up_lpp3070-KanR-down_lpp3070	Sequential cloning at pUC18 (ThermoFisher): (1) Insert from PCR of 1kb region upstream of *lpp3070* with oligos 2676 and 2677, digested with *Hind*III and *Sal*I; (2) Insert from PCR of kanamycin resistance cassette from plasmid pKD4-kanR with oligos 2678 and 2679, digested with *Sal*I and *Kpn*I; (3) Insert from PCR of 1kb region downstream of *lpp3070* with oligos 2680 and 2681, digested with *KpnI* and *Eco*RI	This work
pIF467	pUC18::up_lpp1450-KanR-down_lpp1450	Sequential substitution at pIF463: (1) Insert from PCR of 1kb region upstream of *lpp1450* with oligos 2733 and 2734, digested with *Hind*III and *Sal*I; (2) Insert from PCR of 1kb region downstream of *lpp3070* with oligos 2735 and 2736, digested with *KpnI* and *Eco*RI	This work
pIF485	pUC18::up_vfx10045-KanR-down_vfx10045	Sequential substitution at pIF463: (1) Insert from PCR of 1kb region upstream of *vfx10045* with oligos 2846 and 2847, digested with *Hind*III and *Sal*I; (2) Insert from PCR of 1kb region downstream of *vfx10045* with oligos 2848 and 2849, digested with *KpnI* and *Eco*RI	This work

TABLE S3. Oligonucleotides used in this work.

Name	Sequence (5'→3')
1822	AATTCATGTACCCATACGATGTTCCAGATTACGCTTACCCATACGATGTTCCAGATTACGCTTACCCATACGATGTTCCAGATTACGCTTACC CATACGATGTTCCAGATTACGCTGGCGCGG
1823	GATCCCGCGCCAGCGTAATCTGGAACATCGTATGGGTAAGCGTAATCTGGAACATCGTATGGGTAAGCGTAATCTGGAACATCGTATGGG TAAGCGTAATCTGGAACATCGTATGGGTACATG
1938	AAAAGGATCCATGAAGAAAACTGTGCTGTGGGG
1939	AAAAAAGCTTTTAATTCTGGGTATTTGTTGTAGAGCC
1940	AAAAGGATCCATGACAAGACAAAAGTGGAAAGATC
1941	AAAAAAGCTTTTAAAGTTTCATTTGACTTTCGTTTCC
1942	TTTTGGATCCTTAAGGTTTAAAATCTTTTAATTC
1944	AAAAGGATCCATGGTGGAAAAGAATAAAGATAAAG
1945	TTTTAAGCTTCTAGTTTTTTTTGGCAACCCAAACCG
1948	AAAAGGATCCTTAGCTTGGAGAGTCTGCTTCATG
1949	AAAGGTACCATGAAAAAAATGATCGGTTTTGTG
1950	AAAAGGATCCATGTGTTCAATCACTCACTTTG
1951	AAAAAGCTTTTAATCAGGTACCGGGATCGGACG
1952	AAAAGGATCCTTAAATATTTTTACAAGGTGTCGG
1953	AAAGGTACCATGACGATAGAATGTTATTCTATTG
1954	AAAAGGATCCATGCGTCTTTTGTTTATCAGTTTAATTC
1955	AAAAAAGCTTCTACTGAGGTGATAACAGGGAAG
1956	AAAAGGATCCATGAATTTTTTAAACAATAAAATTG
1957	AAAAAAGCTTTTATCTGCCTTTCCTTGACAAATG
2353	CCATTGATAAAAGCAATCAAGCGTGAAATTGC
2354	GCAATTTCACGCTTGATTGCTTTTATCAATGG
2355	CCAAATTCATCAACAAAAGCCAAAAATAAAAAACCAGAGTGG
2356	CCACTCTGGTTTTTTATTTTTGGCTTTTGTTGATGAATTTGG
2400	AAAAGGTACCATTCCTTTTGTAATTCGC
2401	AAAAGGATCCTTATTTCCCATTGTCTAGACAGC
2423	GCAGCCGCAGCTGCAGCACCAGAGTGGCATTTAAAATTCTTTAATG
2424	TGCTGCAGCTGCGGCTGCTGTTGATGAATTTGGTGATTTATC
2426	AAAAGGATCCTTATTCCGTATTAATACCATATCG
2427	AAAAGGATCCTTAGGATTTCCATGGAATTTTGC
2428	AAAAGGATCCTTATTGAGAGCATAAGCTTTTCTG
2429	AAAAGGATCCTTAAATTCCCTTTGAAGTTTTGC
2430	AAAAGGATCCTTATTGAGCGACATCCTGAGC
2445	AAAAGGTACCATGACAAAACAAAAGTGG
2446	AAAATCTAGACTAATTGAGCTTCATATGG
2447	AAAATCTAGATTAAATATTTTTGCAAAGAGTCGG
2456	AAAACTCGAGTTTCCCATTGTCTAGACAGC
2457	AAAACTCGAGATTTTTAATCATCGATATGG
2484	AAAACTCGAGCAATTGCGTTCCTGCAAATTC
2485	AAAACTCGAGTCCCAACCCGAGAAAAAAATTATC
2486	AAAACTCGAGGAAGGCAACCTTTATAAATTTTTTG
2495	AAAAGGTACCTCCCAACCCGAGAAAAAAATTATC
2533	AAAAGGTACCATGGACTACAAGGACCACGACGGTG
2534	AAAAGGTACCTCCCAACCTGAGAAAAAAAATACC
2535	AAAAGGATCCTTATTGAGCAATATCCTTAACTTTC
2552	AAAACTGCAGAAATGACGATAGAATGTTATTCTATTG
2553	AAAAGGTACCGGATTTCCATGGAATTTTGC
2602	AAGCGGCCGCAAAATATTTTTACAAGGTGTCGG
2604	GGGGGGATCCAATGTCATGACGATAGAATGTTATTCTATTG
2622	AAAGGATCCGCCACCATGGCAATGACGATAGAATGTTATTCTATTGATGGTG
2623	AAGGTACCGCCACCATGGCAGAACAAAAACTCATCTCAGAAGAGGATCTGATGACGATAGAATGTTATTCTATTGATGGTG
2676	GATCAAGCTTGGATATGGCAGATAACGG
2677	GATCGTCGACGGTTGAGAAAGACTTAAGG
2678	GATCGTCGACGGATGAATGTCAGCTACTG
2679	GATCGGTACCCCCAACCTTTCATAGAAGG
2680	GATCGGTACCCTTATATACTTTCGCATCGC
2681	GATCGAATTCCTTTGATGGCAGCGATATC
2733	GATCAAGCTTGAGATTATATTGGCAACTTCCG
2734	GATCGTCGACCTTTCCGAGTTATACTATCTAAACC
2735	GATCGGTACCAGAAGCTATTTTTAGATTGCCAGG
2736	GATCGAATTCGGCGCATCAATAAATGGATACAC
2737	GATCTCTAGACTAATTGAGCTTCATGTGGTTTTC
2738	CTCTGAATTATTTACTCCAATGACTAACTCCTTTCACTC
2739	TGGAGTAAATAATTCAGAGGG
2774	GCAGGAACGCAATTGAGAGCATAAG
2790	GCAAAACTTCAAAGGGAATTTTTAATC
2791	GATTAAAAATTCCCTTTGAAGTTTTGC
2809	GCAAATTCTTTGTCTCAAGCGTTAACAATGCAG
2810	CTGCATTGTTAACGCTTGAGACAAAGAATTTGC
2811	CCCAGCAGAGCCAGTAACAATGAATATTAC
2812	GTAATATTCATTGTTACTGGCTCTGCTGGG
2829	GCAAATTCTTTGTCTCCGCAATTAACAATGCAG
2830	CTGCATTGTTAATTGCGGAGACAAAGAATTTGC
2832	CTGCATTGTTAGCCCTGGAGACAAAGAATTTGC
2835	GCAAATTCTTTGTCTCCAGGGCTAACAATGCAG

2846	GATCAAGCTTCCAACGAAATAAAATGACAACCC
2847	GATCGTCGACTGTTTTGGCAATATGTTATTGTGTG
2848	GATCGGTACCCTTTACATCCACTTTCTCATTACC
2849	GATCGAATTCATATATGGCTTTGGCTACTTCTC
2850	CAAGCTTGCATGCCTGCAG
2852	AAGCGGCCGCAAAATATTTTTGCAAAGAGTCGG
